# Supplementary material for: Synergistic impacts of global warming and thermohaline circulation collapse on amphibians
Source: Commun Biol. 2021 Jan 29;4:141. doi: 10.1038/s42003-021-01665-6 (PMC7846744; doi:10.1038/s42003-021-01665-6)
Supplement: Supplementary file 2 — Supplementary Information [file 42003_2021_1665_MOESM2_ESM.pdf]

Synergistic impacts of global warming and thermohaline circulation collapse on amphibians

Julián A. Velasco[1], Francisco Estrada[1,2,3*], Oscar Calderón-Bustamante[1], Didier Swingedouw[4], Carolina Ureta[1], Carlos Gay[1], Dimitri DeFrance[5,6]

1. Centro de Ciencias de la Atmosfera, Universidad Nacional Autónoma de México, 04510, CDMX, Mexico.

2. Institute for Environmental Studies, VU Amsterdam, 1081 HV, Amsterdam, the Netherlands.

3. Programa de Investigación en Cambio Climático, Universidad Nacional Autónoma de México, 04510, CDMX, Mexico.

4. Environnements et Paléoenvironnements Océaniques et Continentaux, CNRS, Université de Bordeaux, 33615 Pessac, France

5. ESPACE-DEV, Univ Montpellier, IRD, Univ Guyane, Univ Reunion, Univ Antilles, Univ Avignon, Maison de la Télédétection, 500 rue Jean-François Breton, F-34093, Montpellier, Cedex, France.

6. The Climate Data Factory, 75012 Paris, France

*Corresponding author.

Email: feporrua@atmosfera.unam.mx

Supplementary Information

Contents

S1. Biogeographical realms
S2. Description and analysis of climatic and bioclimatic variables
S2.1 Description of control and hosing climate experiments
S2.2 Differences between scenarios and time horizons
S2.3 Climatic anomalies and novel climates in thermohaline circulation weakening scenarios
S3. Ecological modelling approach and extended results
S3.1 Selection of ecological niche modeling algorithms
S3.2 Estimates of range contractions using ecological niche modelling algorithms
S3.3 Range contractions across most diverse taxonomic groupings and extinction risk status
S3.4 Potential effect of uncertainty in model algorithm, threshold criteria and grain size
S3.5 Comparisons of projected range contractions between scenarios and time horizons
S3.6 Geographical patterns of amphibian species losses

S1. Biogeographical realms

We selected endemic amphibian species from six biogeographical realms (Figure S1; see main text). These geographical delimitations are used by biogeographers and macroecologists to organize global diversity.

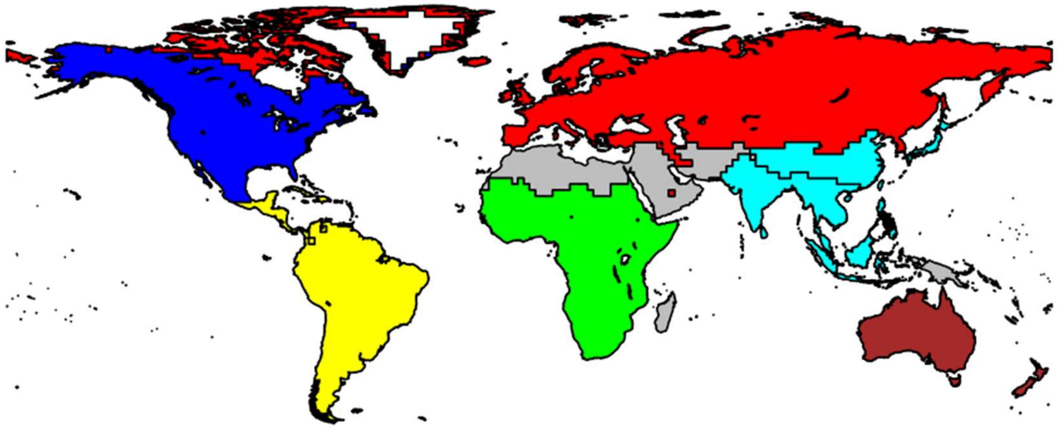

Figure S1. Biogeographical realms (in color) used in this paper. Colors as follow: blue: Nearctic; yellow: Neotropical; green=Afrotropical; brown=Australian; cyan: Indomalayan; red: Palearctic.

S2. Description and analysis of climatic and bioclimatic variables

S2.1 Description of control and hosing climate experiments

The climate simulations used in this study were produced with the *Institut Pierre Simon Laplace* low-resolution coupled ocean-atmosphere model (IPSL-CM5-LR)[1]. The spatial resolution of the atmospheric component is 3.75ºx1.875º in longitude and latitude, respectively, and includes 39 vertical levels. The nominal resolution of the oceanic component is 2º with a higher latitudinal resolution of 0.5° in the equatorial ocean, and 31 vertical levels. The locations for the release of the freshwater are deep water formation regions in the North Atlantic (45ºN to 65ºN, 45ºW to 5ºE), which are classical regions of spread of the input of Greenland meltwater[2] . Since this freshwater input corresponds to Greenland ice sheet melting in terms of its long-term reservoir of freshwater, there is no reason to compensate for the water mass added, and no compensation is added in the model. Since this is a free surface model, this is not requested for conservation issues.

Five experiments are considered for the assessment presented here: a) the control run is the RCP8.5 emission scenario; b) four hosing experiments that are based on the RCP8.5 but include the addition of 0.11, 0.22, 0.34, and 0.68 Sv (1 Sv = 10^6 m^3/s) of freshwater released in the North Atlantic from 2020 to 2070[3]. These experiments are labeled as A, B, C, and D, respectively. All simulations cover the period 2006-2100. Figure S2 illustrates the additional weakening of AMOC over the century that is produced by each of these hosing scenarios. The bioclimatic indices used in this paper are derived from the monthly temperature and precipitation data produced with the IPSL-CM5-LR.

Early studies about the effects of an AMOC collapse were based on climate models' simulations in which hosing experiments were imposed to pre-industrial climate conditions[4] (i.e., other forcing factors such greenhouse gases emissions were held constant). While this type of experiment can help to analyze effects such as climate feedbacks in isolation, a collapse of AMOC is not to be expected to occur under pre-industrial climate or without significant external forcing. In this paper, we use recent simulations in which the hosing experiments are imposed in the course of a high-emissions scenario (RCP8.5)[3]. A high-warming scenario such as the RCP8.5 provides a consistent baseline scenario for exploring the impacts of significant melting of Greenland that produces additional and substantial weakening or collapse of AMOC. Although the probability of a collapse of AMOC is not known, the literature strongly suggests that these probabilities are expected to increase with the level of warming[5,6]. The RCP8.5 represents a high-emission scenario and not a 'business-as-usual' scenario[7]. However, a recent study has shown that this scenario is in close agreement with historical cumulative CO^2 emissions, as well as with projections out to midcentury under current and stated policies, and is characterized by highly plausible levels of CO2 emissions in 2100[8]. We do not assign any probability of occurrence to the RCP8.5 scenario nor to the collapse of AMOC, instead we center on investigating the impacts on amphibian species this climate catastrophe could have.

The assessment of impacts in this paper focuses on climate and thus 30-year averages centered around 2030, 2050 and 2070 are used to represent climate conditions. The effects of climate variability including oscillations such as El Niño/Southern Oscillation (ENSO) and the North Atlantic Oscillation (NAO), or multidecadal climate variations are not

addressed in the analysis and may require the use of large ensembles which are not available at the moment.

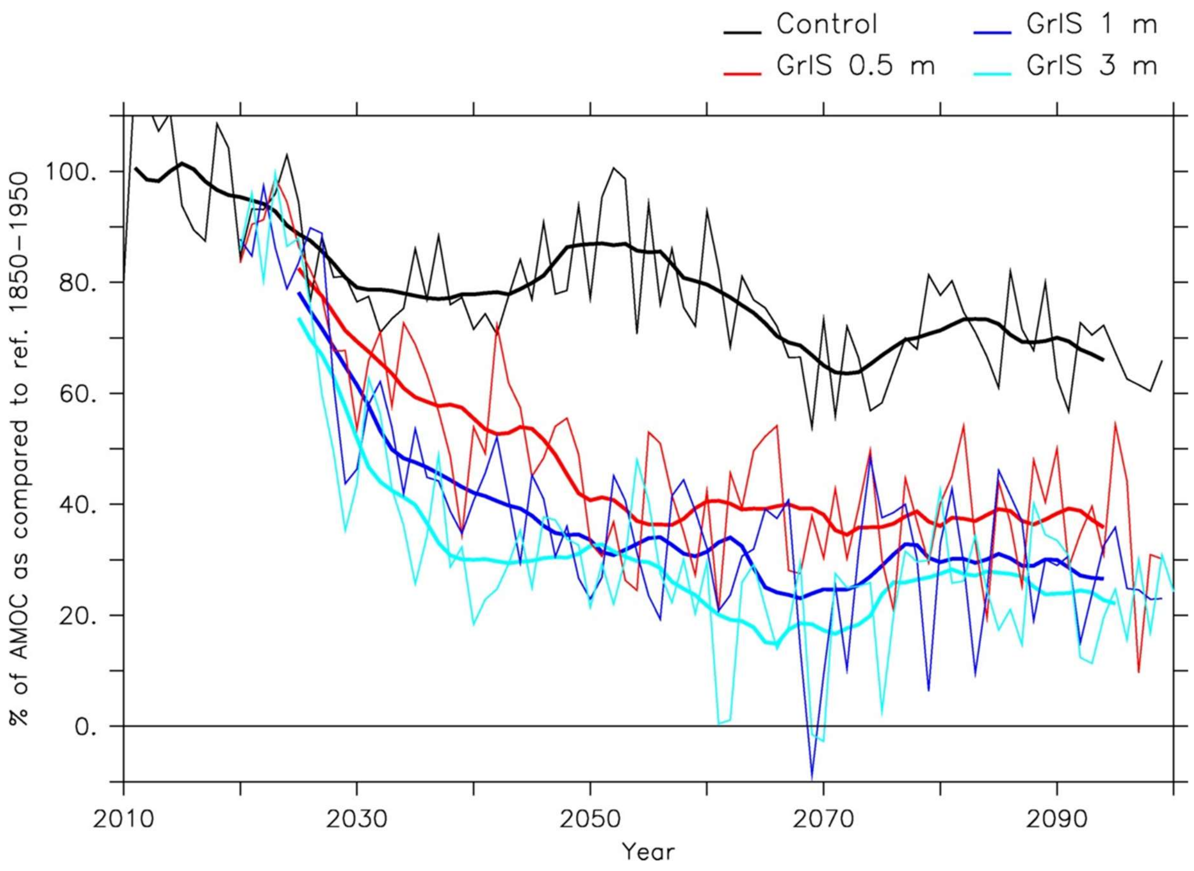

Figure S2. Atlantic meridional overrunning circulation (AMOC) index at 26°N. The AMOC index is defined as the maximum of the Atlantic meridional stream function at 26°N below 500 m. The time evolutions of the indices are shown in percent, in reference to the mean state of the AMOC in the IPSL-CM5A-LR historical simulation over the period 1850-1950. The black line shows the control simulation based on the RCP8.5 projection, while the red, blue and light blue refer to the superimposed hosing experiments with the addition of 0.11, 0.22 and 0.68 Sv of freshwater from 2020 to 2070, respectively (1 Sv = 10^6 m^3/s). Thick and thin lines represent 30-year moving averages and annual frequency data, respectively.

S2.2 Differences between scenarios and time horizons

Bioclimatic variables are more biologically meaningful than precipitation and temperature variables alone and thus are commonly used in ecological modelling. We constructed five bioclimatic variables used to model amphibian species' distributions and that have been considered important for amphibian's biology in previous studies[9]: Annual mean temperature (AMT; bio1); warmest month temperature (WMT; bio5); coldest month temperature (CMT; bio6); total annual precipitation (AP; bio12); and precipitation seasonality (PS; bio15). These bioclimatic variables have a strong influence on species' distributions and functional traits[9–12] and have been used to model species' distributional areas both under current[9] and future climate change conditions[12,13]. Part I of Figures S3 to S7 shows maps of these bioclimatic variables under the control scenario and all the hosing experiments described in S2.1 for the 2070 period, while Part II shows the differences between the control (RCP8.5) and each one of the hosing experiments.

Figure S8 shows radial graphs of the median change for each region, bioclimatic variable and horizon (2030, 2050, 2070), with respect to the reference climatology (1970-2000). Departures from symmetry in each axis denote differences between the RCP8.5 and the RCP8.5 plus additional and substantial weakening of AMOC (experiment D) (e. Some salient features of this figure illustrate the effects of additional and substantial weakening over the climate of the selected regions. For instance, under the RCP8.5 scenario median changes in AMT, CMT and WMT tend to be similar in magnitude and sign for all regions. However, with the additional weakening of the AMOC important contrasts between changes in CMT and WMT appear in all regions and large differences in precipitation with respect to the RCP8.5 control simulation tend to occur.

Moreover, the variation of deltas across scenarios illustrates important non-stationarities across geography in the departure of current climate conditions in the hosing experiments (Figure S9-S14). For instance, CMT and WMT exhibit large departures in all freshwater discharge scenarios with respect to the control simulation. These figures illustrate the degree of climatic departure from baseline conditions for each of the five bioclimatic variables used in the ecological niche models (Figure S9- S14). The variation of these departures is context-dependent and some regions would be more affected by temperature whereas other by changes in rainfall regimes. As denoted by the boxplots in these figures, all bioclimatic variables show very large variability within regions. The delta values from these figures (Figure S9- S14) represent the differences between a given future scenario (e.g., control and hosing experiments) and baseline conditions (1970-2000). The non-stationarity across time and space shows the complexity of these abrupt climate changes and of their potential impacts on ecosystems. For instance, the largest departures were observed in precipitation variables (AP and PS) across all six realms (Figure S9- S14). The departure of extreme monthly temperatures (CMT and WMT) from baseline conditions is context-dependent and in some regions is larger (e.g., Palearctic) than in others (e.g., Neotropical). As it is discussed in the following subsection, large heterogeneities in climatic departures from current conditions are also present within regions which also contribute to the large spatial variability in the projected impacts on biodiversity.

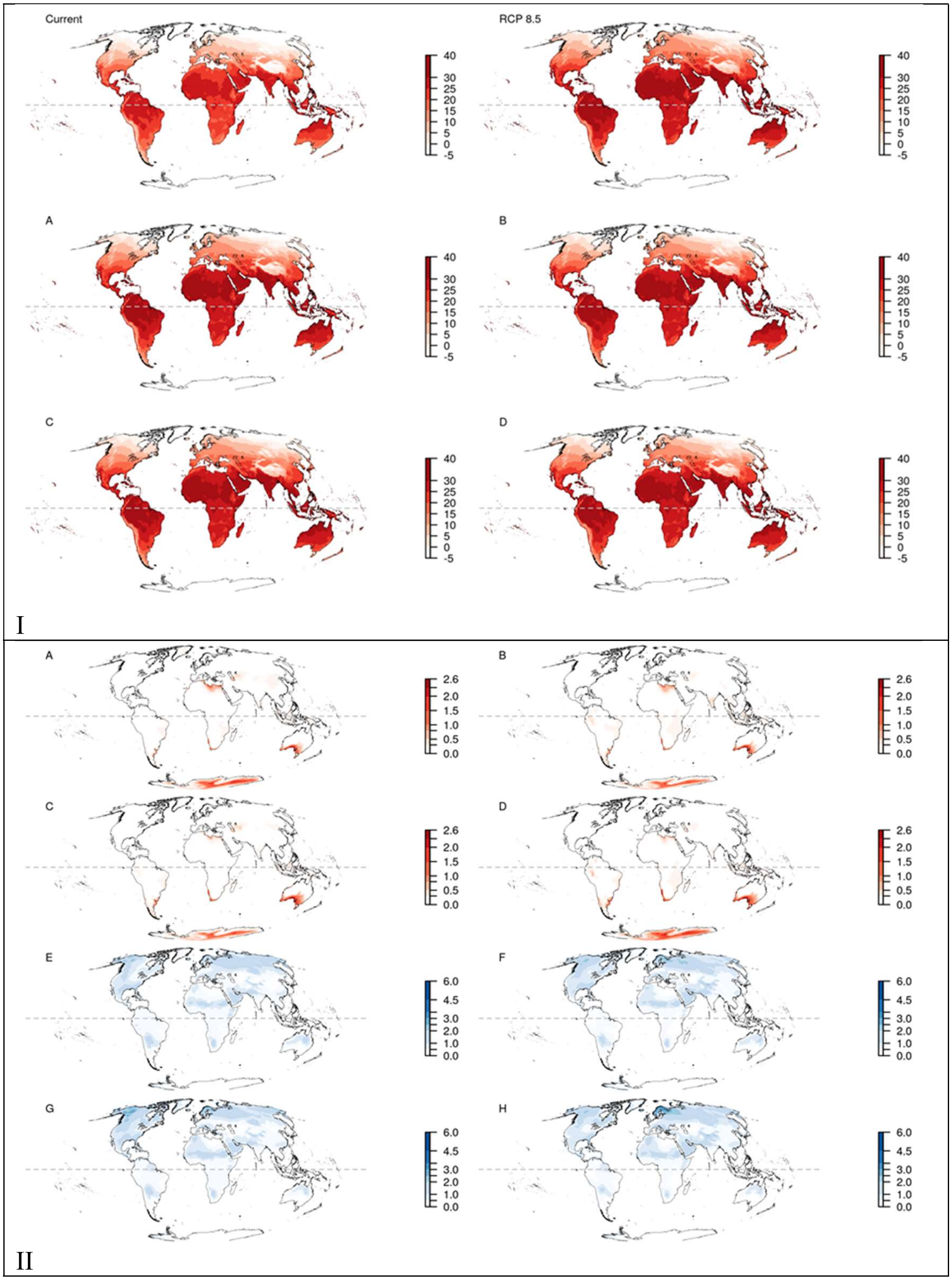

Figure S3. Annual temperature (AMT; ºC) under the control scenario (RCP85) and the hosing experiments. Part I of this figure shows in the upper row the annual temperature values for current conditions (left) and the control reference scenario (right). The middle row shows the annual temperature values for the hosing experiments A (left) and B (right). The lower row

shows the annual temperature values for the hosing experiments C (left) and D (right). Part II shows the differences between the control reference scenario (RCP8.5) and each one of the hosing experiments for 2070 for AMT. The first two rows (A-D) show the anomalies with increases in temperature (i.e., warming) for each one of the hosing experiments (A: Sv=0.11; B: Sv=0.22; C: Sv=0.34; D: Sv=0.68). The last two rows (E-H) show the anomalies with decreases in temperature (i.e., cooling) for each one of the hosing experiments (E: Sv=0.11; F: Sv=0.22; G: Sv=0.34; H: Sv=0.68).

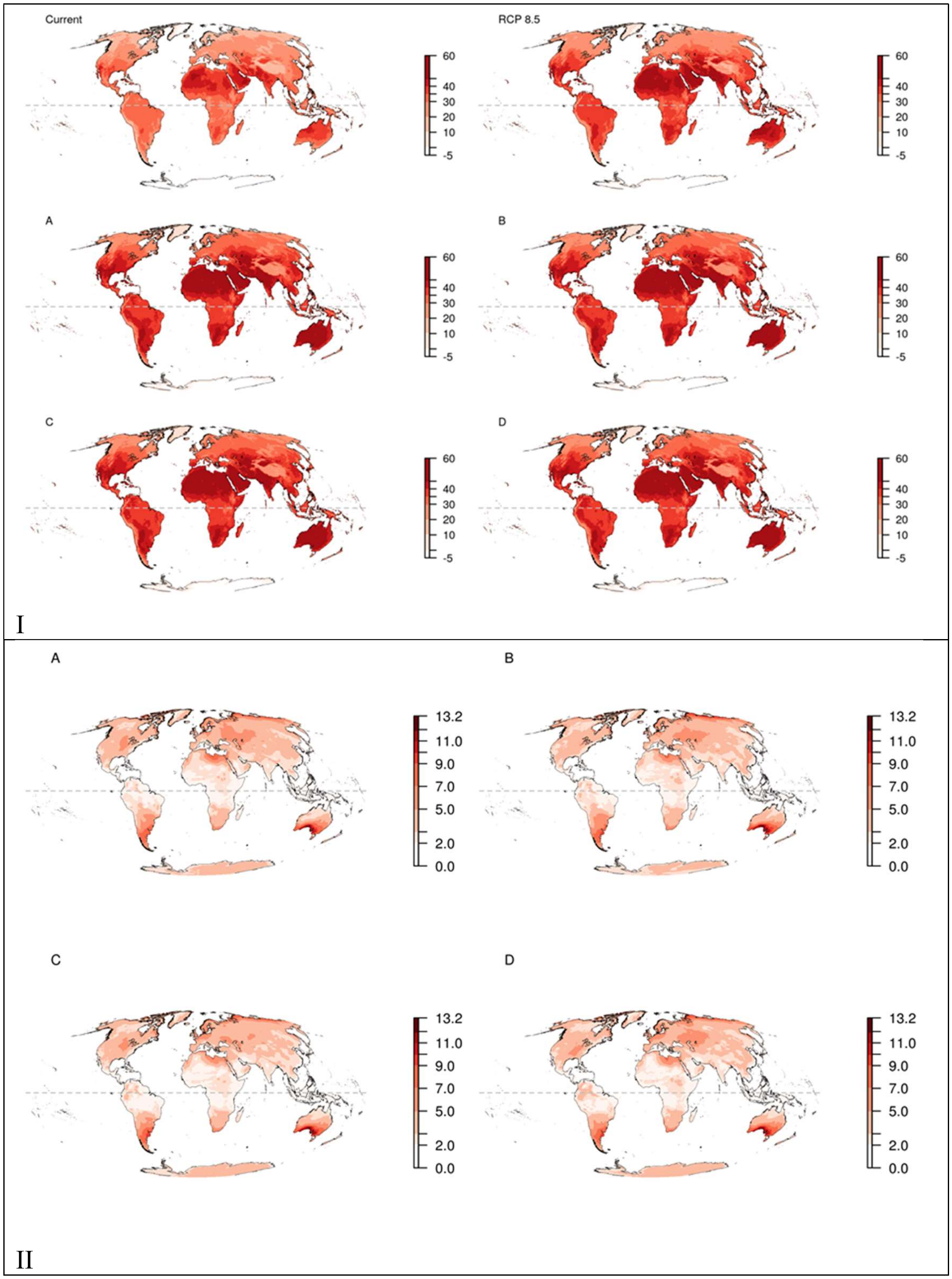

Figure S4. Temperature of the warmest month (WMT; ºC) under the control reference scenario (RCP85) and the hosing experiments. Part I of this figure shows in the upper row the temperature values of the warmest month for current conditions (left) and the control scenario (right). The middle row shows the temperature values of the warmest month for the hosing experiments A (left) and B (right). The lower row shows the temperature values of

the warmest month for the hosing experiments C (left) and D (right). Part II shows the differences between the control reference scenario (RCP85) and each one of the hosing experiments for 2070 for WMT. The plots (A-D) shows the anomalies with increases in temperature (i.e., warming) for each one of the hosing experiments (A: Sv=0.11; B: Sv=0.22; C: Sv=0.34; D: Sv=0.68). The anomalies with decreases in temperature (i.e., cooling) were restricted to a few pixels and therefore are not shown.

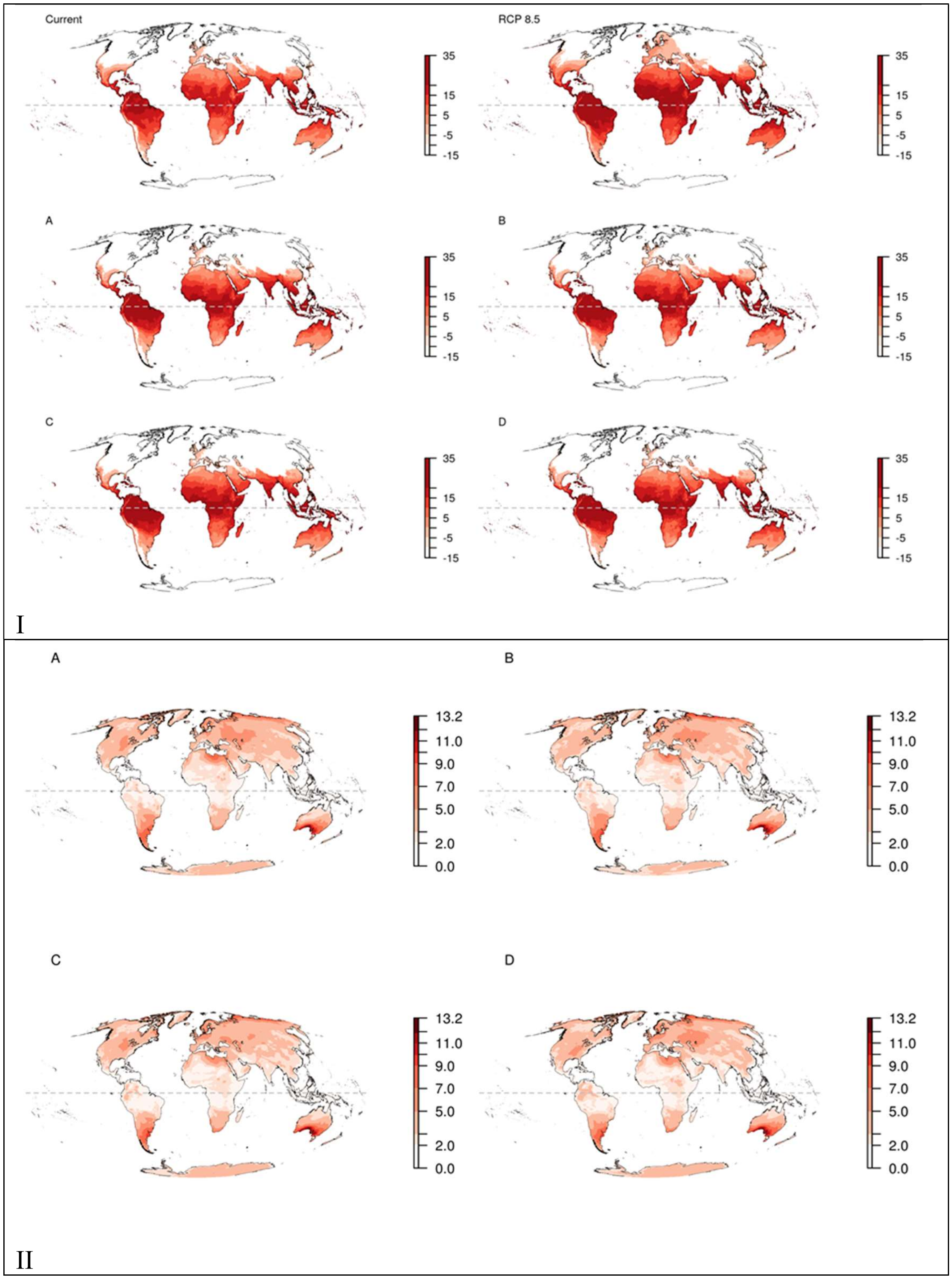

Figure S5. Temperature of the coldest month (CMT; ºC) under the control reference scenario (RCP85) and the hosing experiments. Part I of this figure shows in the upper row the temperature values of the coldest month for current conditions (left) and the control scenario (right). The middle row shows the temperature values of the coldest month for the hosing

experiments A (left) and B (right). The lower row shows the temperature values of the coldest month for the hosing experiments C (left) and D (right). Part II shows the differences between the control scenario (RCP85) and each one of the hosing experiments for 2070 for CMT. The plots (A-D) shows the anomalies with increases in temperature (i.e., warming) for each one of the hosing experiments (A: Sv=0.11; B: Sv=0.22; C: Sv=0.34; D: Sv=0.68). The anomalies with decreases in temperature (i.e., cooling) were restricted to a few pixels and therefore are not shown.

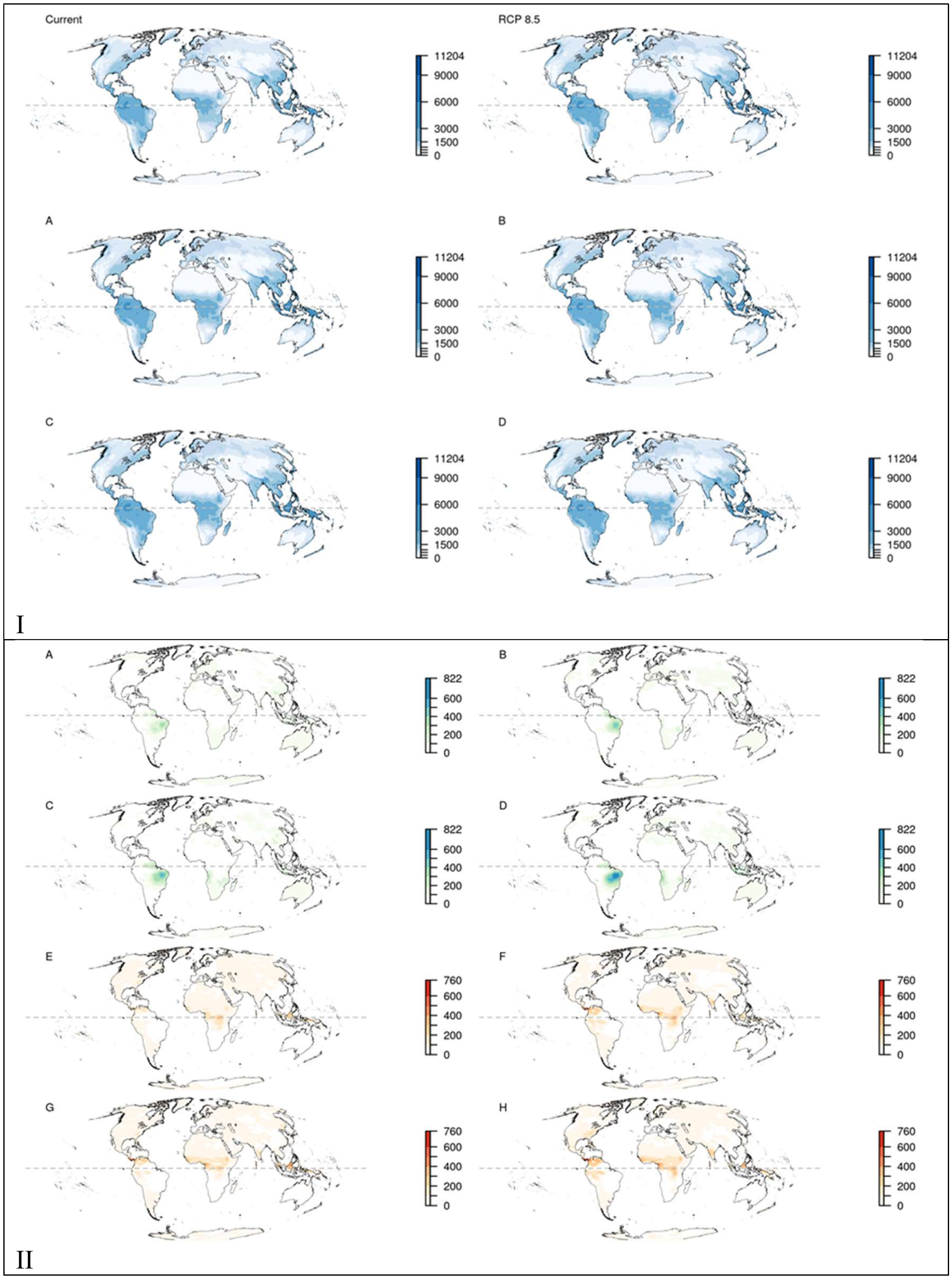

Figure S6. Total annual precipitation (AP; mm) under the control reference scenario (RCP85) and the hosing experiments. Part I of this figure shows in the upper row the total annual precipitation values for current conditions (left) and the control scenario (right). The middle row shows the total annual precipitation values for the hosing experiments A (left) and B (right). The lower row shows the total annual precipitation values for the hosing experiments

C (left) and D (right). Part II shows the differences between the control reference scenario (RCP85) and each one of the hosing experiments for 2070 for AP. The first two rows (A-D) show the anomalies with increases in precipitation (i.e., wetter) for each one of the hosing experiments (A: Sv=0.11; B: Sv=0.22; C: Sv=0.34; D: Sv=0.68). The last two rows (E-H) show the anomalies with decreases in precipitation (i.e., drier) for each one of the hosing experiments (E: Sv=0.11; F: Sv=0.22; G: Sv=0.34; H: Sv=0.68).

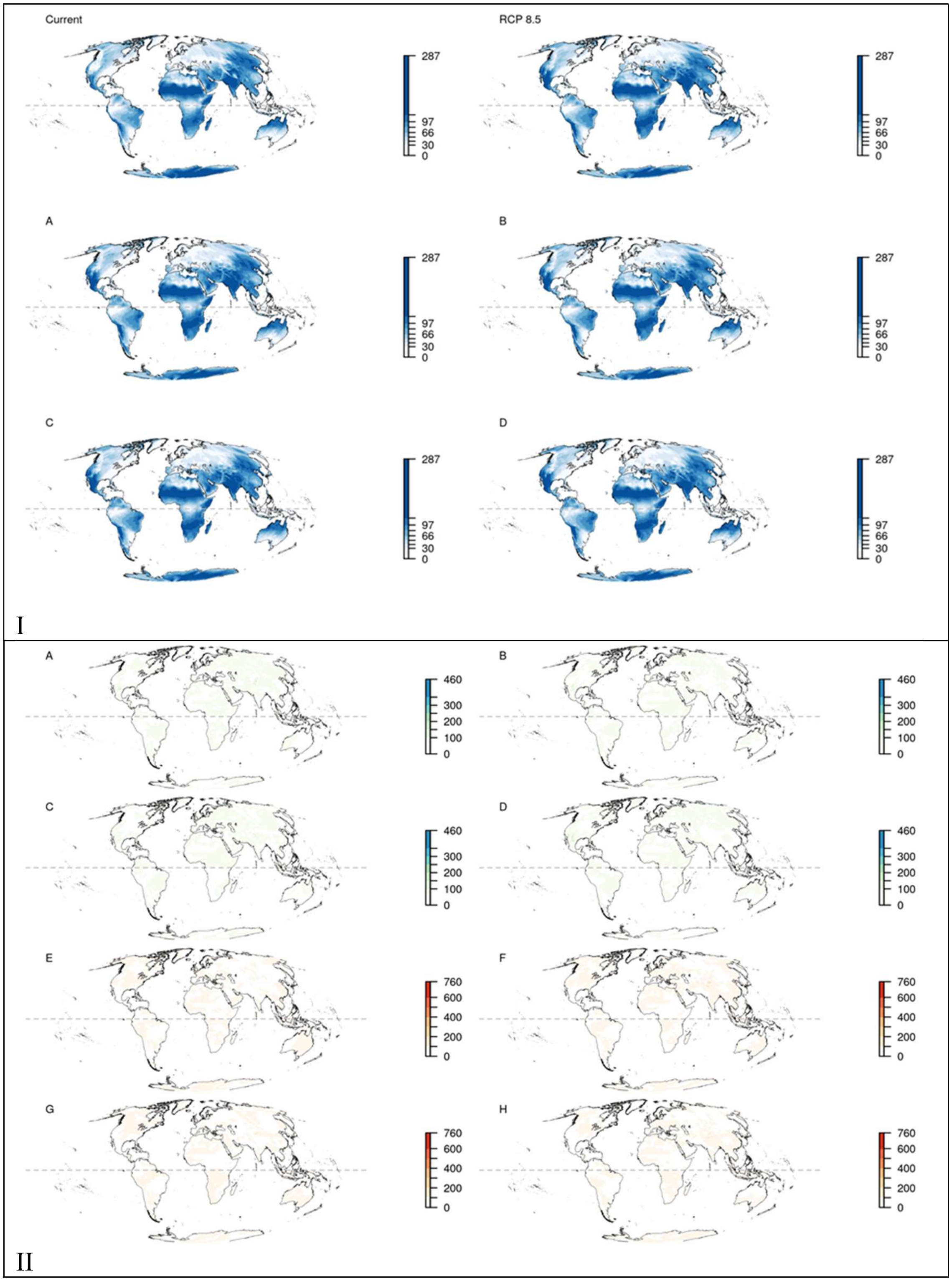

Figure S7. Precipitation seasonality (PS) under the control reference scenario (RCP85) and the hosing experiments. Part I of this figure shows in the upper row the precipitation seasonality values for current conditions (left) and the control scenario (right). The middle row shows the precipitation seasonality values for the hosing experiments A (left) and B (right). The lower row shows the precipitation seasonality values for the hosing experiments

C (left) and D (right). Part II shows the differences between the control reference scenario (RCP85) and each one of the hosing experiments for 2070 for PS. The first two rows (A-D) show the anomalies with increases in precipitation seasonality for each one of the hosing experiments (A: Sv=0.11; B: Sv=0.22; C: Sv=0.34; D: Sv=0.68). The last two rows (E-H) show the anomalies with decreases in precipitation seasonality for each one of the hosing experiments (E: Sv=0.11; F: Sv=0.22; G: Sv=0.34; H: Sv=0.68).

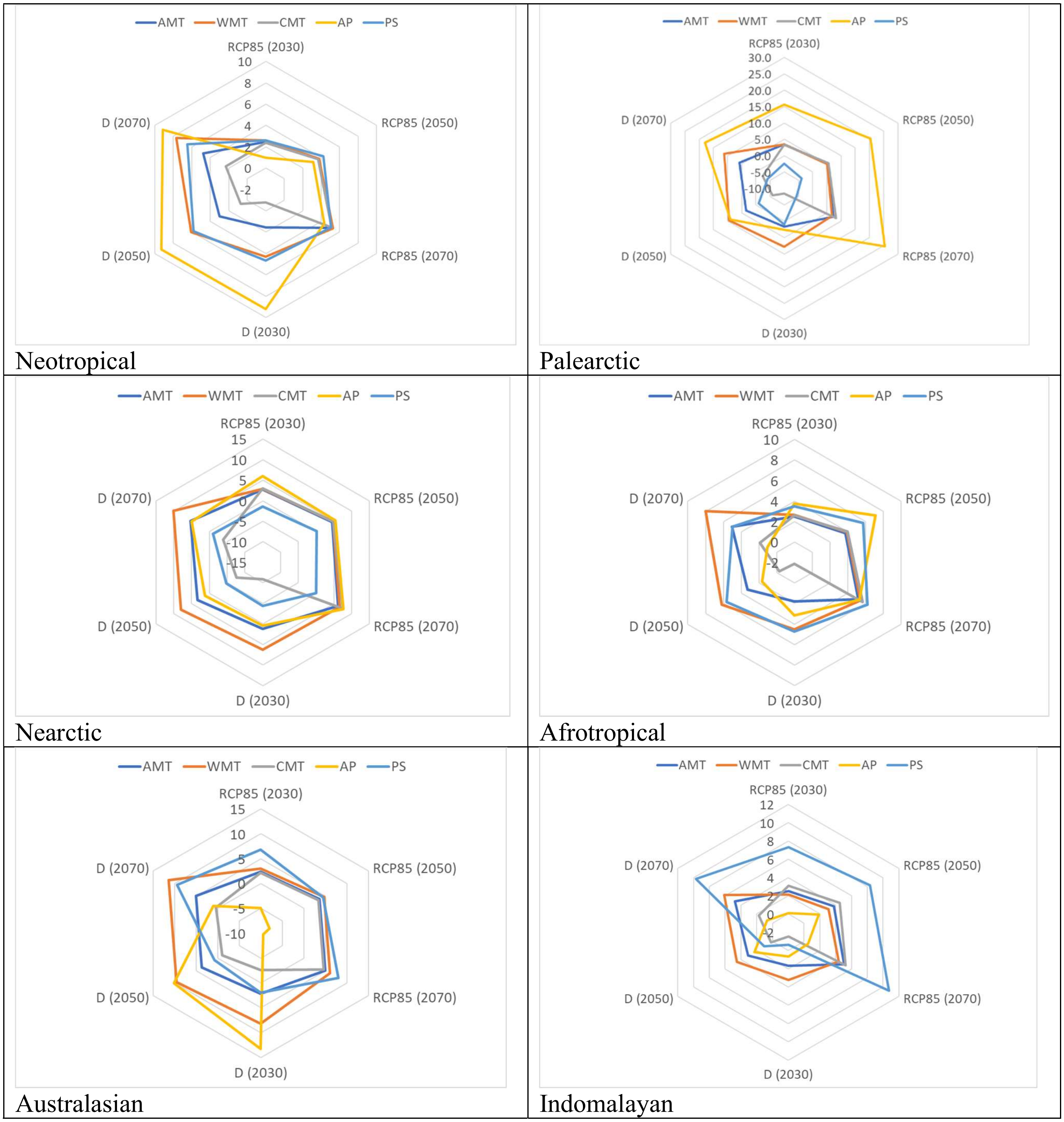

Figure S8. Comparison of the RCP8.5 and the RCP8.5 plus additional and substantial AMOC weakening for the 2030, 2050 and 2070 horizons, and for each region. Departures from symmetry in each axis denote differences between the RCP8.5 and the RCP8.5 plus additional and substantial weakening of AMOC (D, 0.68 Sv). AMT: annual mean temperature; AP: annual precipitation; CMT: coldest month temperature; PS: precipitation seasonality; WMT: warmest month temperature. Variables are expressed as median changes from current conditions and AP is in % units.

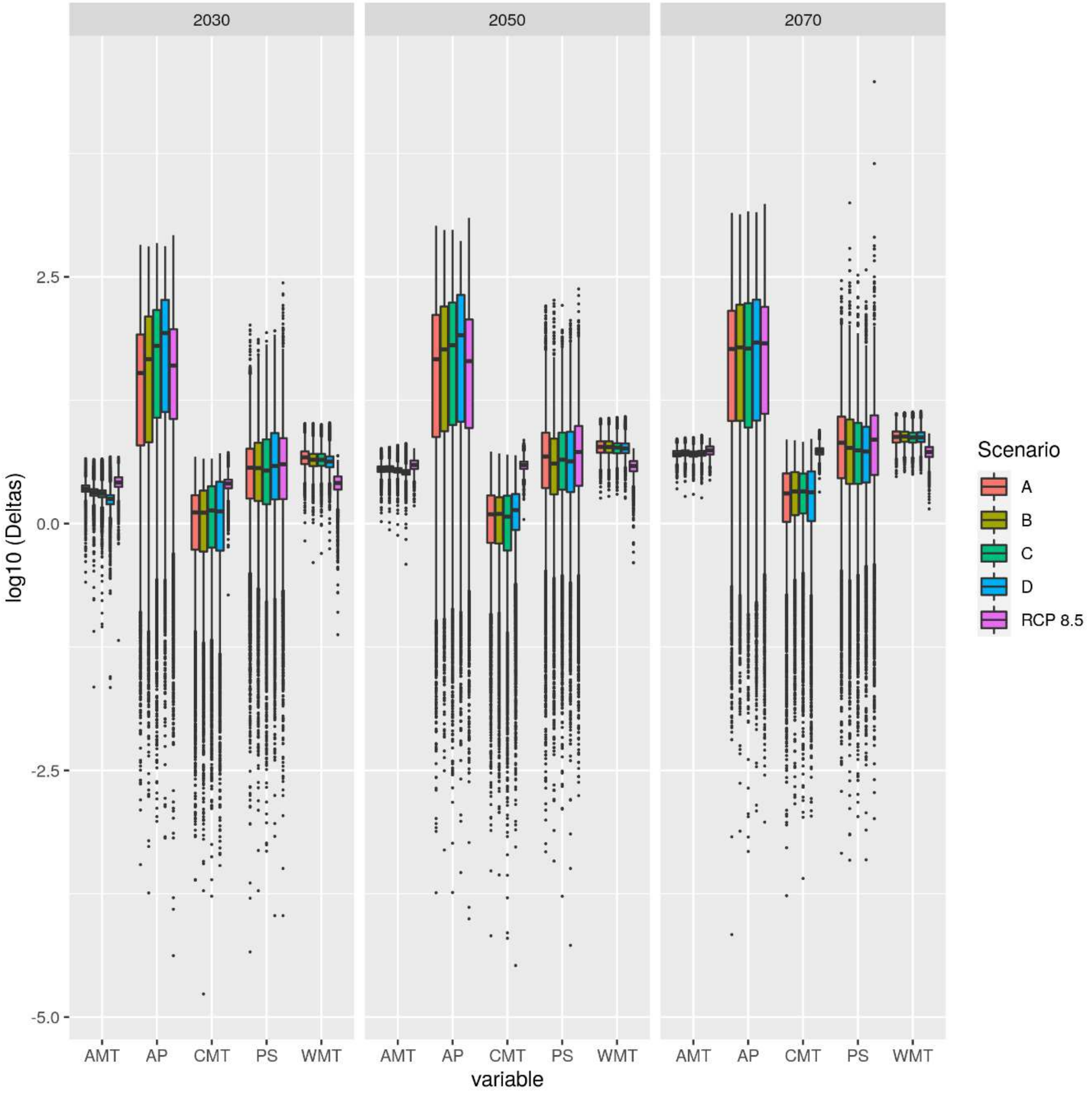

Figure S9. Boxplots illustrating variation in the differences between current climate conditions and each climate change scenario (deltas) for each one of the five bioclimatic variables for the Afrotropical region. AMT: annual mean temperature; AP: annual precipitation; CMT: coldest month temperature; PS: precipitation seasonality; WMT: warmest month temperature.

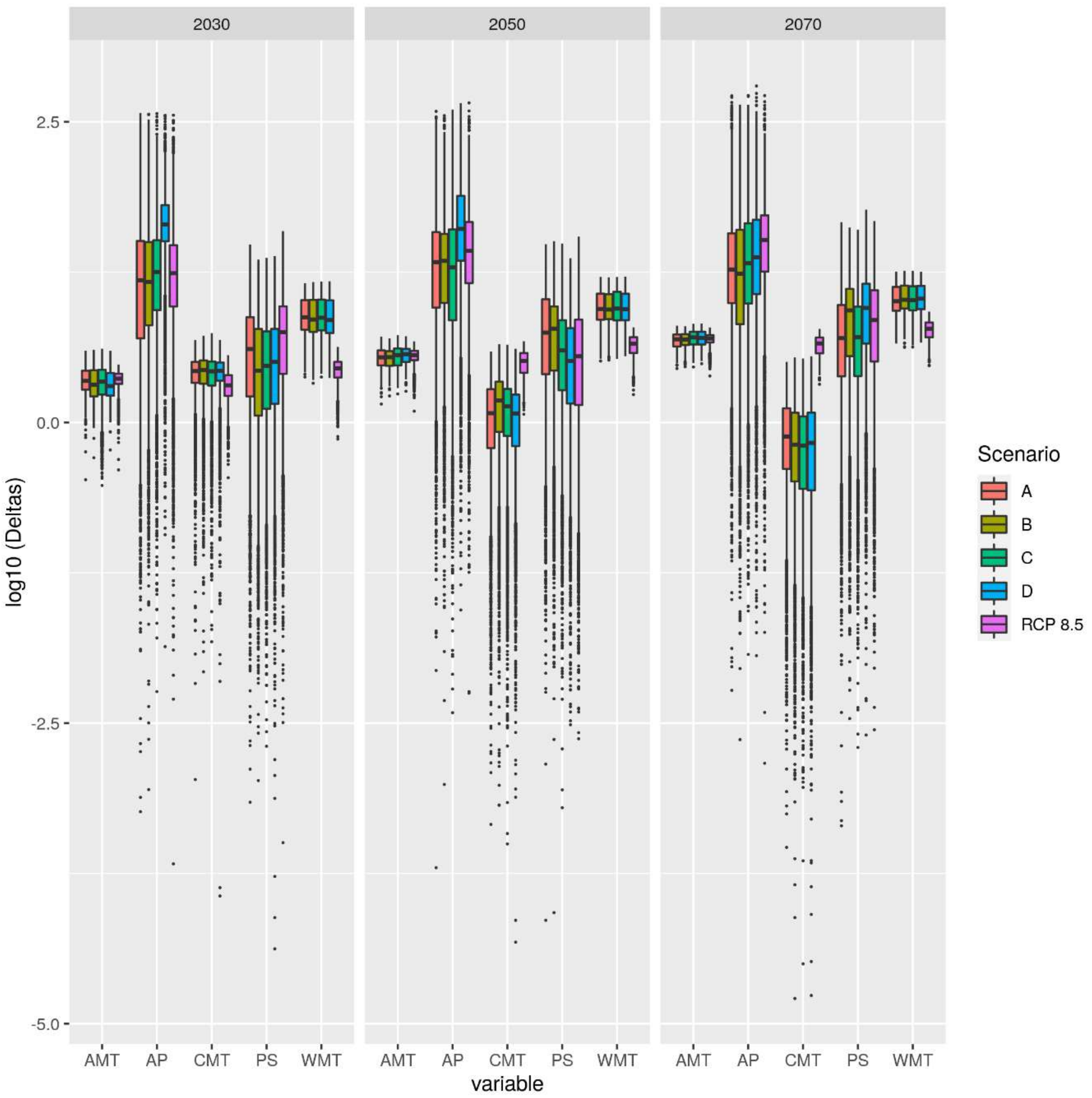

Figure S10. Boxplots illustrating variation in the differences between current climate conditions and each climate change scenario (deltas) for each one of the five bioclimatic variables for the Australasian region. AMT: annual mean temperature; AP: annual precipitation; CMT: coldest month temperature; PS: precipitation seasonality; WMT: warmest month temperature.

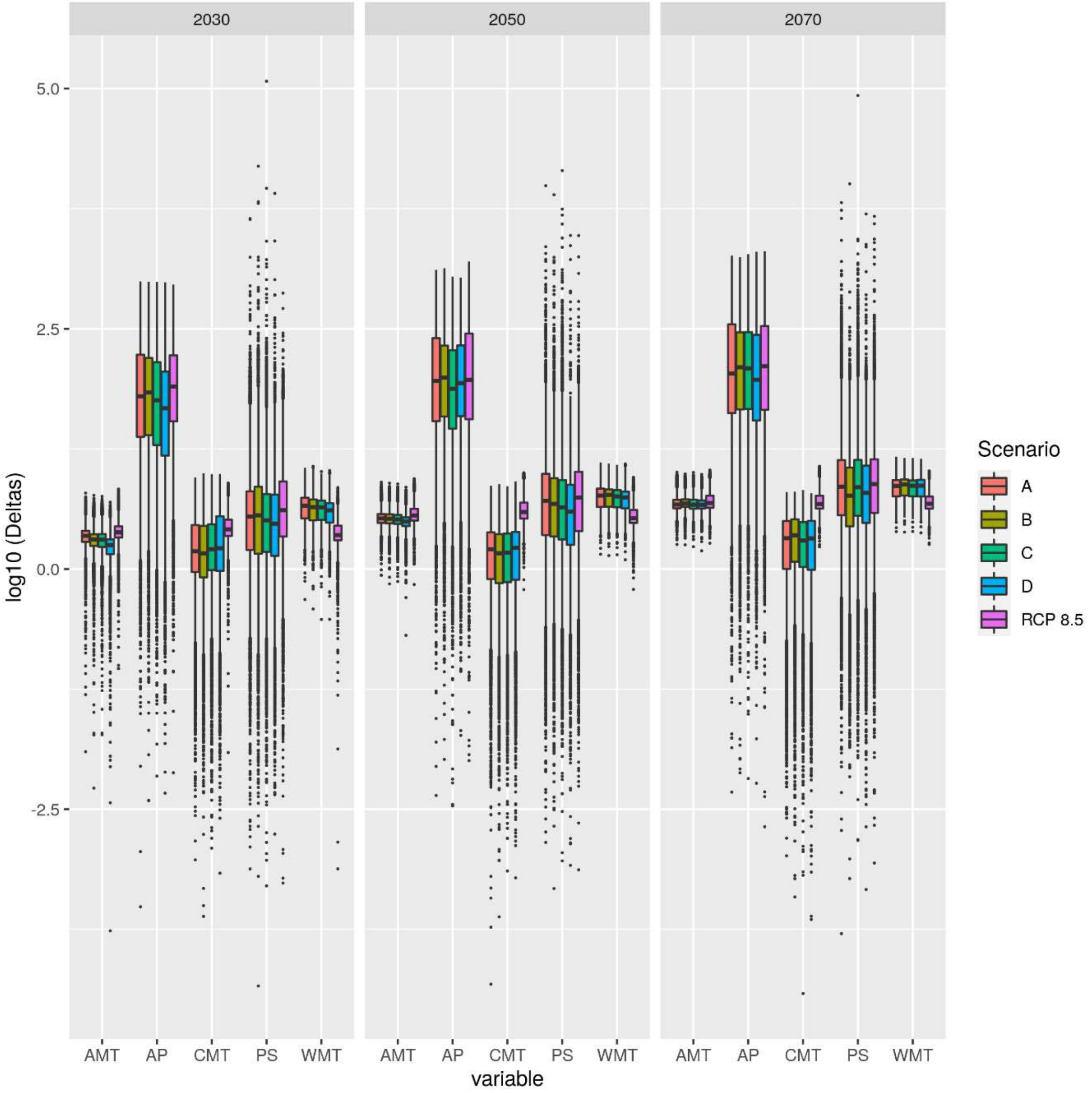

Figure S11. Boxplots illustrating variation in the differences between current climate conditions and each climate change scenario (deltas) for each one of the five bioclimatic variables for the Indomalayan region. AMT: annual mean temperature; AP: annual precipitation; CMT: coldest month temperature; PS: precipitation seasonality; WMT: warmest month temperature.

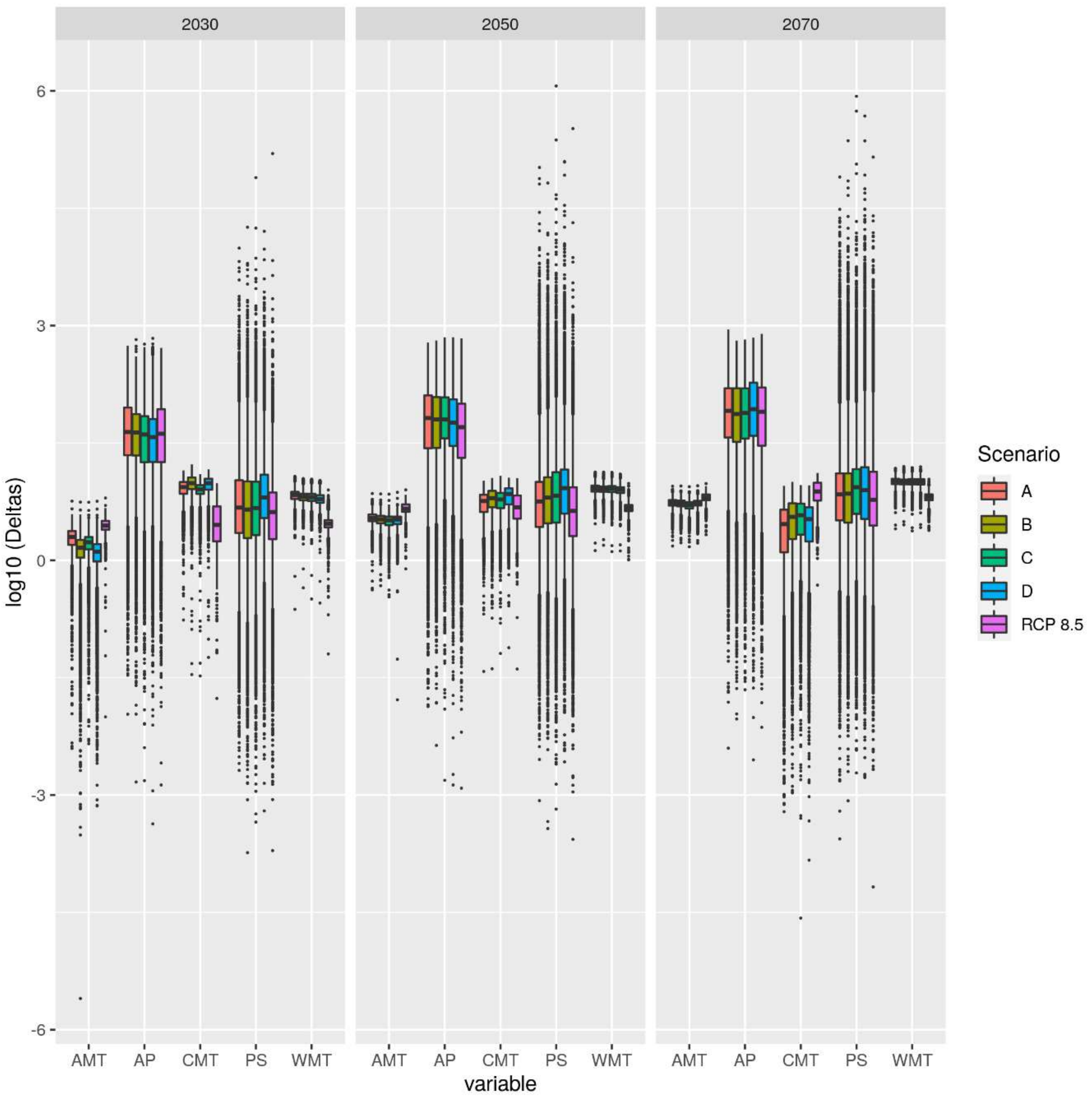

Figure S12. Boxplots illustrating variation in the differences between current climate conditions and each climate change scenario (deltas) for each one of the five bioclimatic variables for the Nearctic region. AMT: annual mean temperature; AP: annual precipitation; CMT: coldest month temperature; PS: precipitation seasonality; WMT: warmest month temperature.

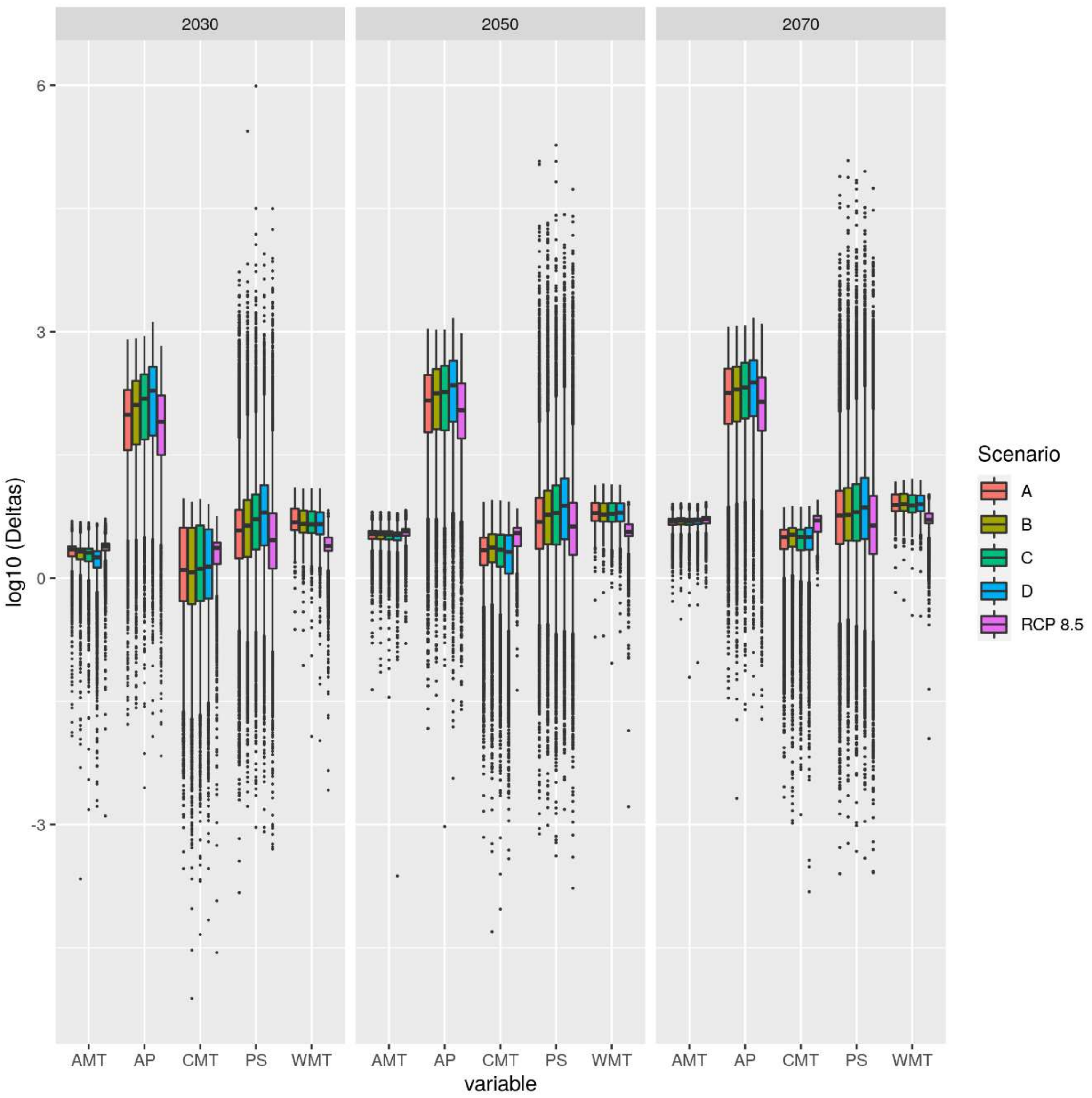

Figure S13. Boxplots illustrating variation in the differences between current climate conditions and each climate change scenario (deltas) for each one of the five bioclimatic variables for the Neotropical region. AMT: annual mean temperature; AP: annual precipitation; CMT: coldest month temperature; PS: precipitation seasonality; WMT: warmest month temperature.

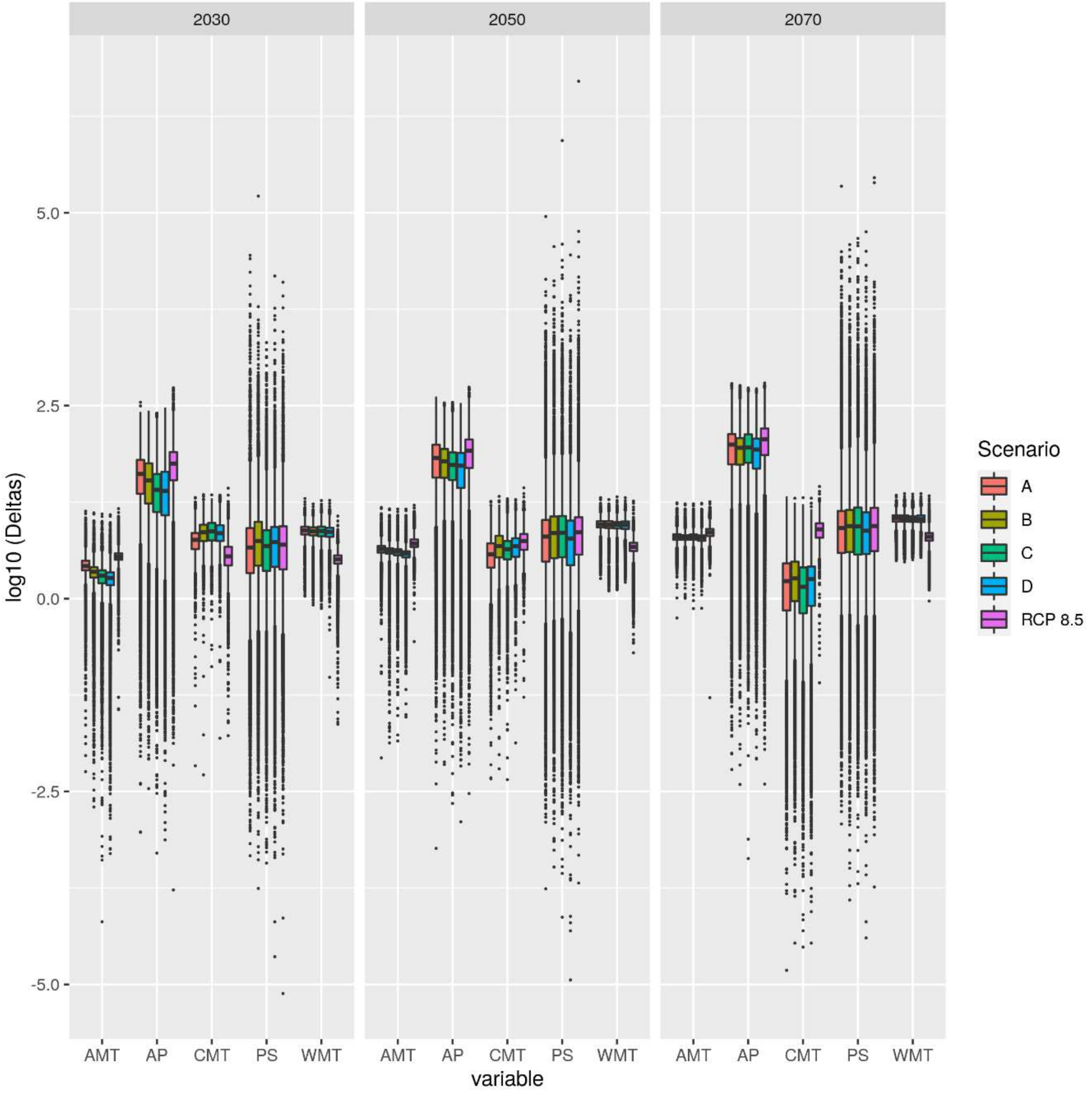

Figure S14. Boxplots illustrating variation in the differences between current climate conditions and each climate change scenario (deltas) for each one of the five bioclimatic variables for the Palearctic region. AMT: annual mean temperature; AP: annual precipitation; CMT: coldest month temperature; PS: precipitation seasonality; WMT: warmest month temperature.

S2.3 Climatic anomalies and novel climates in thermohaline circulation weakening scenarios

We also analyzed the spatial emergence of novel climates (i.e., non-analog climates)[14] and climatic departures based on the set of five bioclimatic variables described above. These analyses were performed for all experiments: the control reference scenario (RCP 8.5) and each of the four hosing experiments (0.11 Sv; 0.22 Sv; 0.34 Sv; 0.68 Sv). Climatic departures were calculated as the Euclidean distances between future climatic conditions and current climatic conditions (Figure S15)[15]. Some regions experience larger departures in the short term (2030; e.g., Australasian, Nearctic and Palearctic) than others (Figure S15). However, Euclidean distances reveal that more extreme climatic conditions could emerge in several regions across time under an AMOC collapse scenario (see outliers in Figure S15). Figure S16 depicts the Euclidean distances at the grid cell level for the control and hosing simulations during the first part of this century (2030). This figure shows that climate departures are much larger under four the hosing experiments than under the control scenario (RCP8.5), particularly for regions such as Neotropical, Nearctic and Palearctic and for the 0.68 Sv scenario.

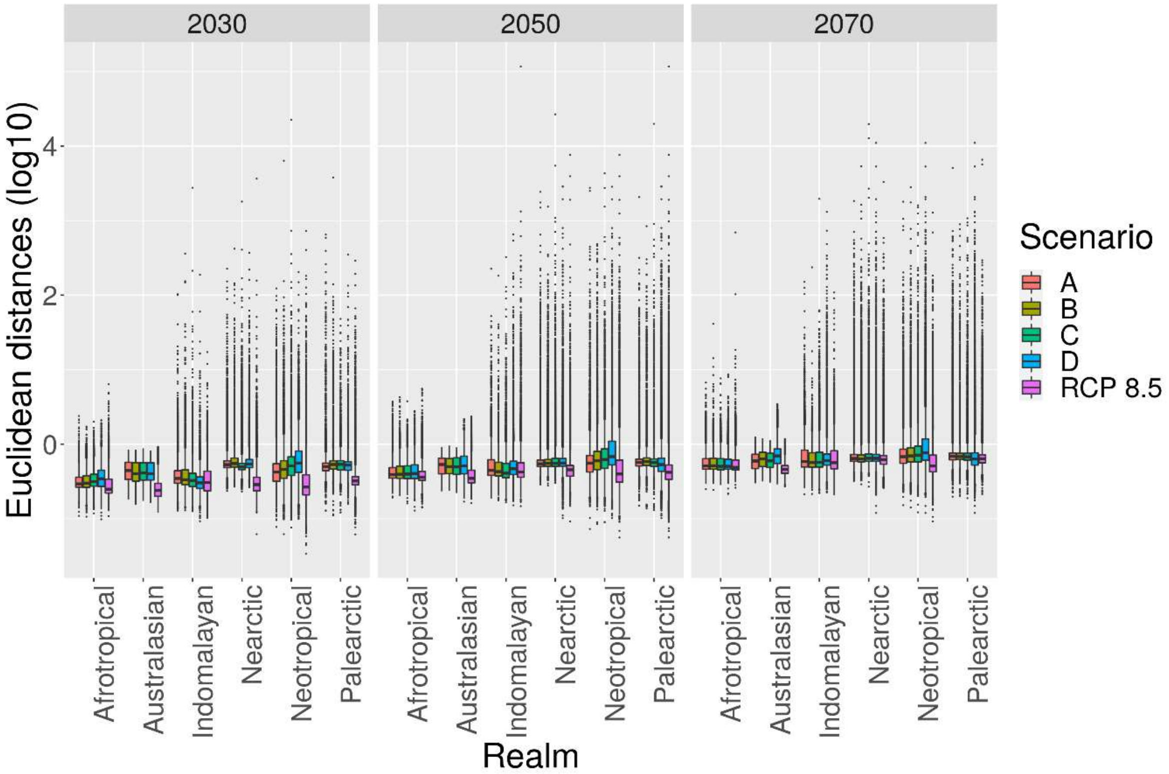

Figure S15. Euclidean distances (log10) based on five bioclimatic variables. Variables are expressed as climatic anomalies between each climate change scenario and current climate conditions.

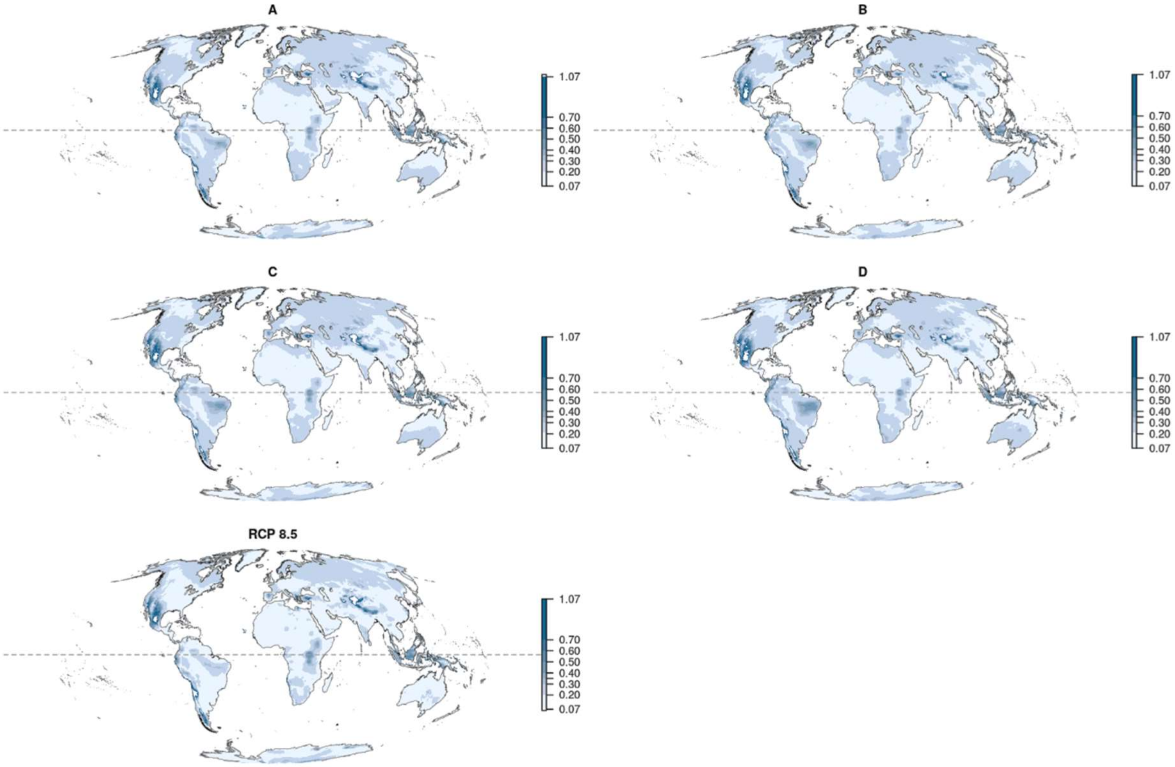

Figure S16. Euclidean distances (log10 based on five bioclimatic variables for the 2030 horizon). Panel a-d shows the Euclidean distances for the control plus freshwater discharge simulations A, B, C and D, respectively. Panel e shows the Euclidean distances for the control scenario (RCP 8.5). Large values indicate climatic combinations very different to current conditions (i.e., novel or non-analog climates).

S3. Ecological modelling approach and extended results

3.1 Selection of ecological niche modeling algorithms

A set of 15 species were selected randomly for each biogeographical realm and species distribution models were fitted using five algorithms: MaxEnt, MARS, CART, ANN, GLM and GBM to explore which algorithms have the best predictive performance (e.g., omission rate, AUC, TSS, and Kappa; Table S1; see also[16,17] for full description of these validation metrics). A full description of each model algorithm can be found in Peterson et al.[18] and Guisan et al.[19]. We selected only three algorithms (MaxEnt; CART; and BRT) that have a good model performance based on the lowest omission rate, high AUC, and TSS values and a low omission rate. Species distribution models for 2509 species were estimated with these three algorithms. All models show a high predictive performance (high TSS, specificity and sensitivity values; Figure S17). We excluded from subsequent analyses those species with a poor performance denoted by low values in validation metrics (e.g., TSS <= 0.4, sensitivity and specificity <= 0.5).

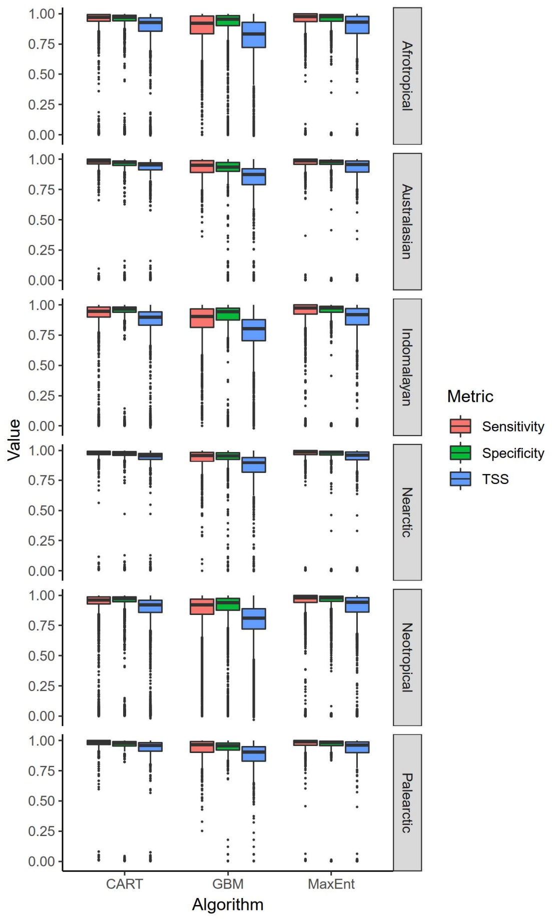

Figure S17. Validation metrics for each algorithm across six biogeographical regions. TSS: True Statistics Skill.

S3.2 Estimates of range contractions using ecological niche modelling algorithms

We performed ecological niche modeling for 2509 endemic species to six biogeographical realms across the world (Table S1). The biogeographical realm with the highest number of modelled species was Neotropical (1211) followed by Afrotropical (466 species). The Palearctic realm had the lowest number of modelled species (108 species).

Table S1. Number of endemic species modelled using ecological niche modelling algorithms.

Region	Endemic species
Afrotropical	466
Australasian	175
Indomalayan	386
Nearctic	163
Neotropical	1211
Palearctic	108

We found an extensive variation in the response of amphibian species to future climate change scenarios. This suggests that amphibian species can respond either positively (i.e, expand its ranges) or negatively (i.e., contract its ranges) to future climate conditions. More species will experience range contractions than range expansions under all climate change scenarios. This result is relatively similar across the three model algorithms (Table S2).

Table S2. Number of species exhibiting range contraction and expansion under future climate change conditions. Species were modelled using three algorithms (MaxEnt: maximum entropy; CART: classification and regression trees; BRT: boosted regression trees) with the highest predictive performance in preliminary modeling runs. Models were transferred to a high-warming scenario (IPLS RCP 8.5), four hosing experiments (HE 0.11, HE 0.22, HE 0.34, HE 0.68 Sv: 1 Sv $=10^6$ m^3/s) of freshwater release in the North Atlantic from 2020 to 2100, and three temporal horizons (2030, 2050, 2070). The column labeled as percentage denotes the percentage of species that contracted their distributional areas.

**Algorithm**	**Scenario**	**2030**			**2050**			**2070**		
		**Contraction**	**Expansion**	**Percentage**	**Contraction**	**Expansion**	**Percentage**	**Contraction**	**Expansion**	**Percentage**
**MaxEnt**	**RCP 8.5**	1967	542	**78**	2015	494	**80**	2027	482	**81**
	**HE 0.11**	2038	471	**81**	2049	460	**82**	2058	451	**82**
	**HE 0.22**	2036	473	**81**	2056	453	**82**	2068	441	**82**
	**HE 0.34**	2035	474	**81**	2034	475	**81**	2044	465	**81**
	**HE 0.68**	2031	478	**81**	2041	468	**81**	2063	446	**82**
**CART**	**RCP 8.5**	1291	984	**57**	1319	956	**58**	1314	961	**58**
	**HE 0.11**	1459	816	**64**	1428	847	**63**	1410	865	**62**
	**HE 0.22**	1456	819	**64**	1453	822	**64**	1415	860	**62**
	**HE 0.34**	1466	809	**64**	1423	852	**63**	1421	854	**62**
	**HE 0.68**	1515	760	**67**	1477	798	**65**	1414	861	**62**
**BRT**	**RCP 8.5**	955	1482	**39**	1059	1378	**43**	1162	1275	**48**
	**HE 0.11**	1250	1187	**51**	1247	1190	**51**	1242	1195	**51**
	**HE 0.22**	1241	1198	**51**	1256	1183	**51**	1260	1177	**52**
	**HE 0.34**	1432	1005	**59**	1339	1098	**55**	1351	1086	**55**
	**HE 0.68**	1297	1140	**53**	1282	1155	**53**	1274	1163	**52**

Furthermore, the percentage of range contractions was relatively similar across biogeographical realms (Table S3). In Nearctic and Palearctic realms, we found that more species contract their ranges across all the hosing scenarios in comparison with a control scenario (IPLS-RCP 8.5). This suggests that endemic amphibians in extra-tropical realms seem to be more sensitive to potential contractions in their distributional areas with climate change scenarios simulating a thermohaline weakening.

Table S3. Percentage of species suffering range contractions across biogeographical realms in a high-warming scenario (RCP 8.5) and four hosing experiments (0.11, 0.22, 0.34, 0.68 Sv; 1 Sv = 10^6 m^3/s) of freshwater release in the North Atlantic from 2020 to 2100), modelled using a maximum entropy algorithm.

**Realm**	**Scenario**	**2030**	**2050**	**2070**
Afrotropical	RCP 8.5	81	82	82
	0.68 Sv	81	84	85
	0.34 Sv	82	83	85
	0.22 Sv	80	85	85
	0.11 Sv	80	83	85
Australasian	RCP 8.5	72	78	75
	0.68 Sv	71	71	73
	0.34 Sv	73	73	74
	0.22 Sv	73	73	75
	0.11 Sv	73	71	73
Indomalayan	RCP 8.5	90	91	91
	0.68 Sv	79	82	87
	0.34 Sv	79	83	86
	0.22 Sv	79	83	87
	0.11 Sv	80	83	83
Nearctic	RCP 8.5	51	57	53
	0.68 Sv	72	73	73
	0.34 Sv	72	72	69
	0.22 Sv	72	71	70
	HE 0.11	72	72	72
Neotropical	RCP 8.5	84	85	87
	0.68 Sv	84	83	84
	0.34 Sv	85	84	84
	0.22 Sv	85	84	84
	0.11 Sv	84	83	84
Palearctic	RCP 8.5	15	18	19
	0.68 Sv	82	72	64
	0.34 Sv	75	72	64

	0.22 Sv	80	74	64
	0.11 Sv	81	70	60

S3.3 Range contractions across most diverse taxonomic groupings and extinction risk status

Range contractions for the most diverse taxonomic groupings and extinction risk status were also estimated for all climate experiments described in section S2.1. Estimated range contractions are large for all taxonomic groupings and risk status even for the 2030 horizon. For the control simulation, the median reduction in distribution range for all taxonomic groupings and risk status is close to 50%, increasing to about 75% by the end of the century (Figures S18, S19). “Critically endangered” and “endangered” show lower range contractions than other categories, particularly when full dispersal is allowed. The range contraction of species is much larger compared to the control scenario across taxonomic groupings (Figure S18), extinction risk status (Figure S19), but not between freshwater discharge levels (Figures 1, S18, S19).

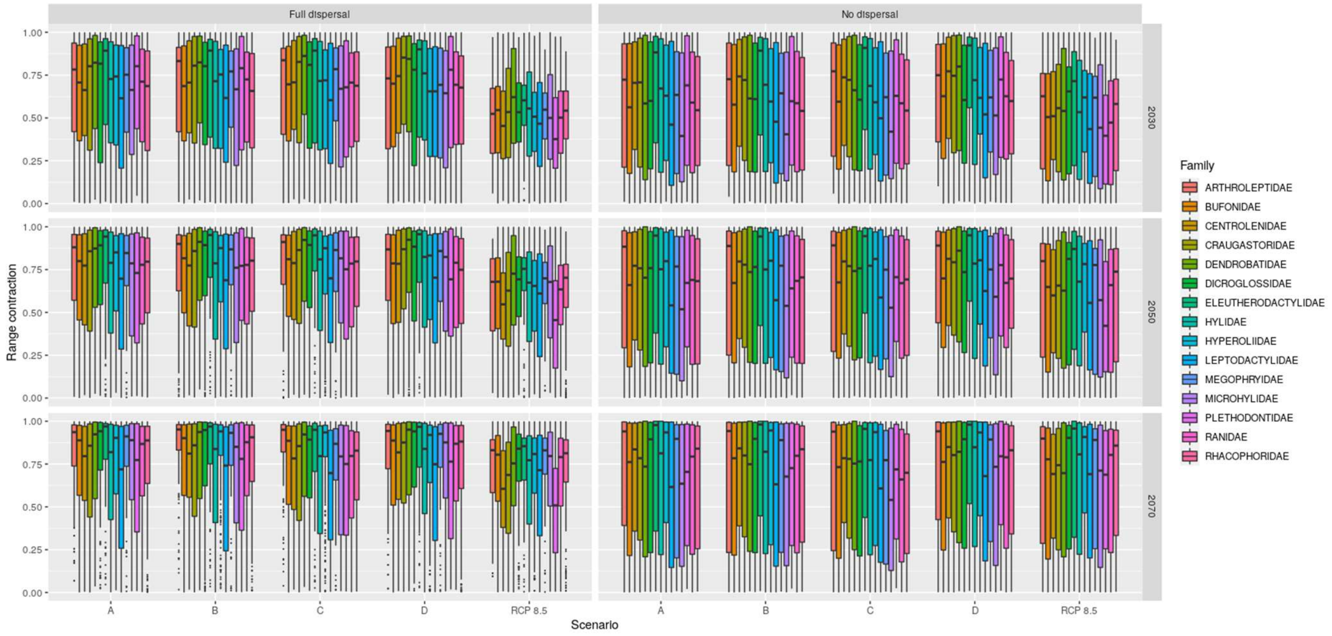

Figure S18. Boxplots of projected range contractions for 2509 amphibian species under a high-warming scenario (IPLS RCP 8.5; reference scenario) and four hosing experiments adding 0.11, 0.22, 0.34, and 0.68 Sv of freshwater (1 Sv = 10^6 m^3/s) labeled as A, B, C, and D, respectively. Range contractions are expressed in proportional terms and are shown for two dispersal scenarios (full dispersal and no dispersal). Results were averaged across three ecological niche modeling algorithms (MaxEnt, BRT, CART; see material and methods) and compared across the most diverse amphibian families (i.e., those with more than 100 species).

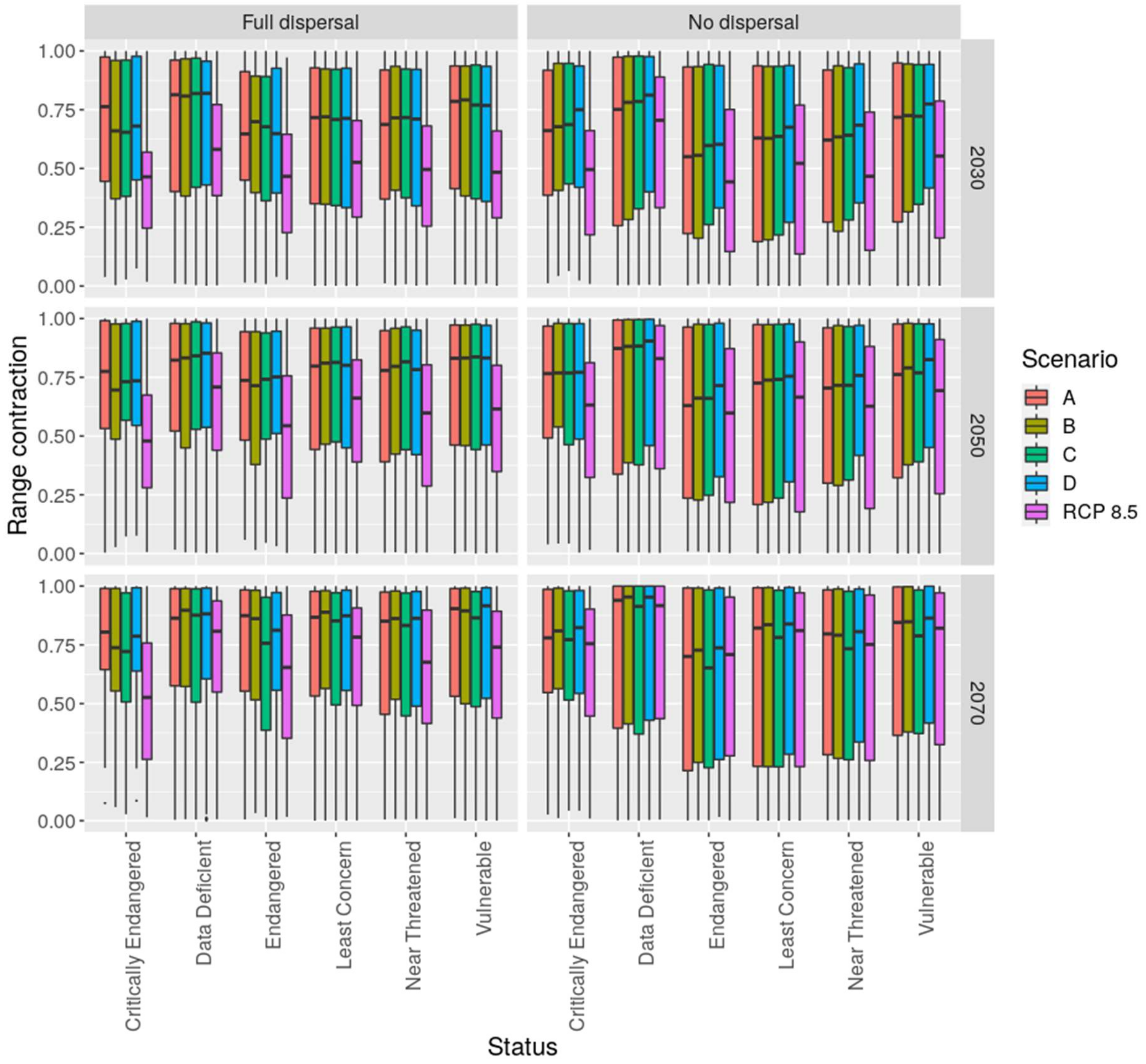

Figure S19. Boxplots of projected range contractions for 2509 amphibian species under a high-warming scenario (IPLS RCP 8.5; reference scenario) and four hosing experiments adding 0.11, 0.22, 0.34, and 0.68 Sv of freshwater (1 Sv = 10^6 m^3/s) labeled as A, B, C, and D, respectively. Range contractions are expressed in proportional terms and are shown for two dispersal scenarios (full dispersal and no dispersal). Results were averaged across three ecological niche modeling algorithms (MaxEnt, BRT, CART; see material and methods) and compared across IUCN extinction risk status.

S3.4 Potential effect of uncertainty in model algorithm, threshold criteria and grain size

Our results are robust to different kinds of uncertainty in projected geographical distributions across a high-warming scenario and four hosing experiments. First, we found that the three algorithms show that, on average, species are projected to experience extensive losses of suitable areas across hosing experiments (Figure S20). We found similar results for the percentage of range contraction, independently of the threshold criteria used (Figure S21). Finally, we did not find differences using a coarser-grain scale (1°) in the patterns of range contraction (Figure S22).

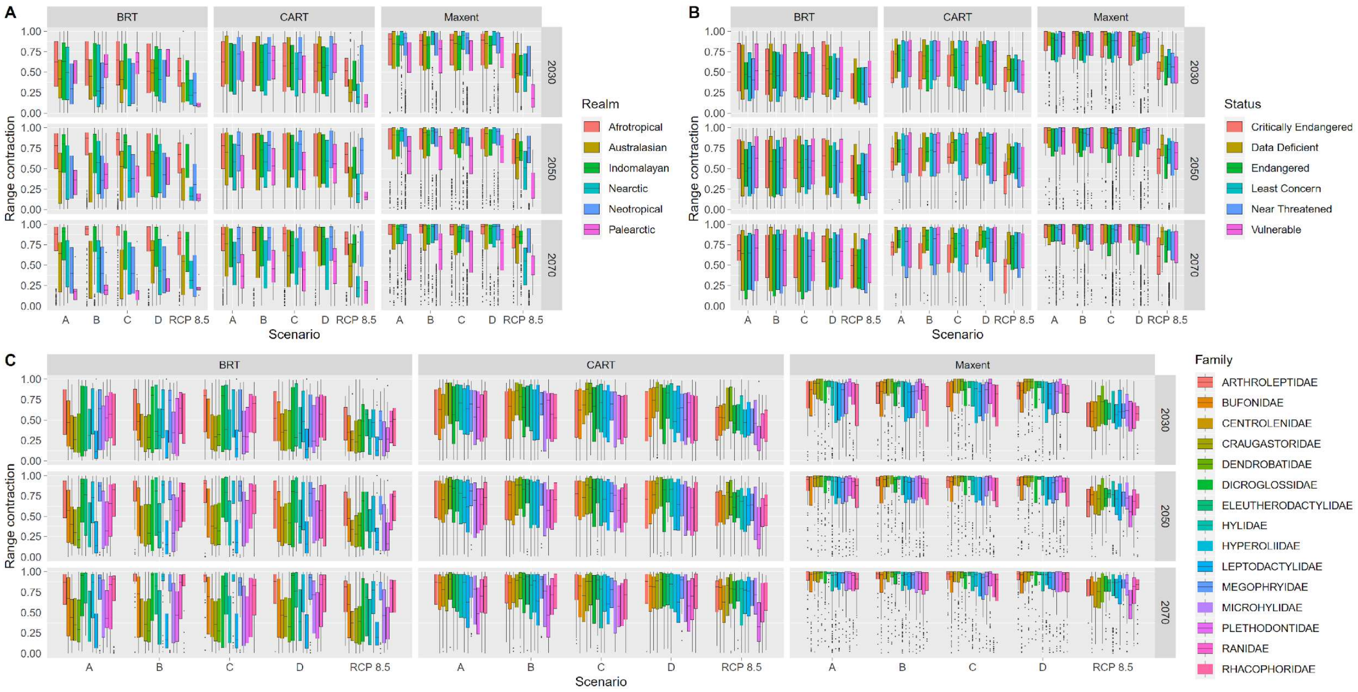

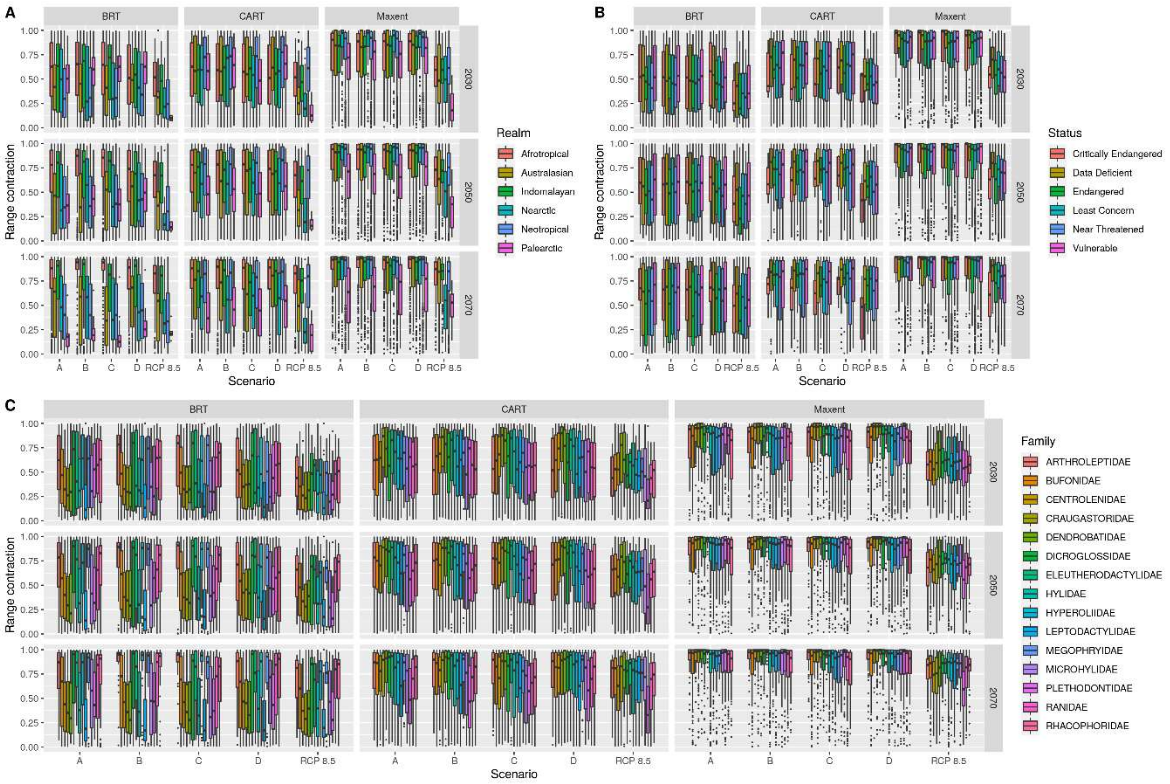

Figure S20. Boxplots of projected range contractions for 2509 amphibian species under a high-warming scenario (RCP 8.5) and four hosing experiments labeled A, B, C, and D, respectively, and three ecological niche algorithms and three time horizons (2030, 2050 and 2070). BRT: boosted regression tree; CART: classification and regression trees; MaxEnt: Maximum entropy.

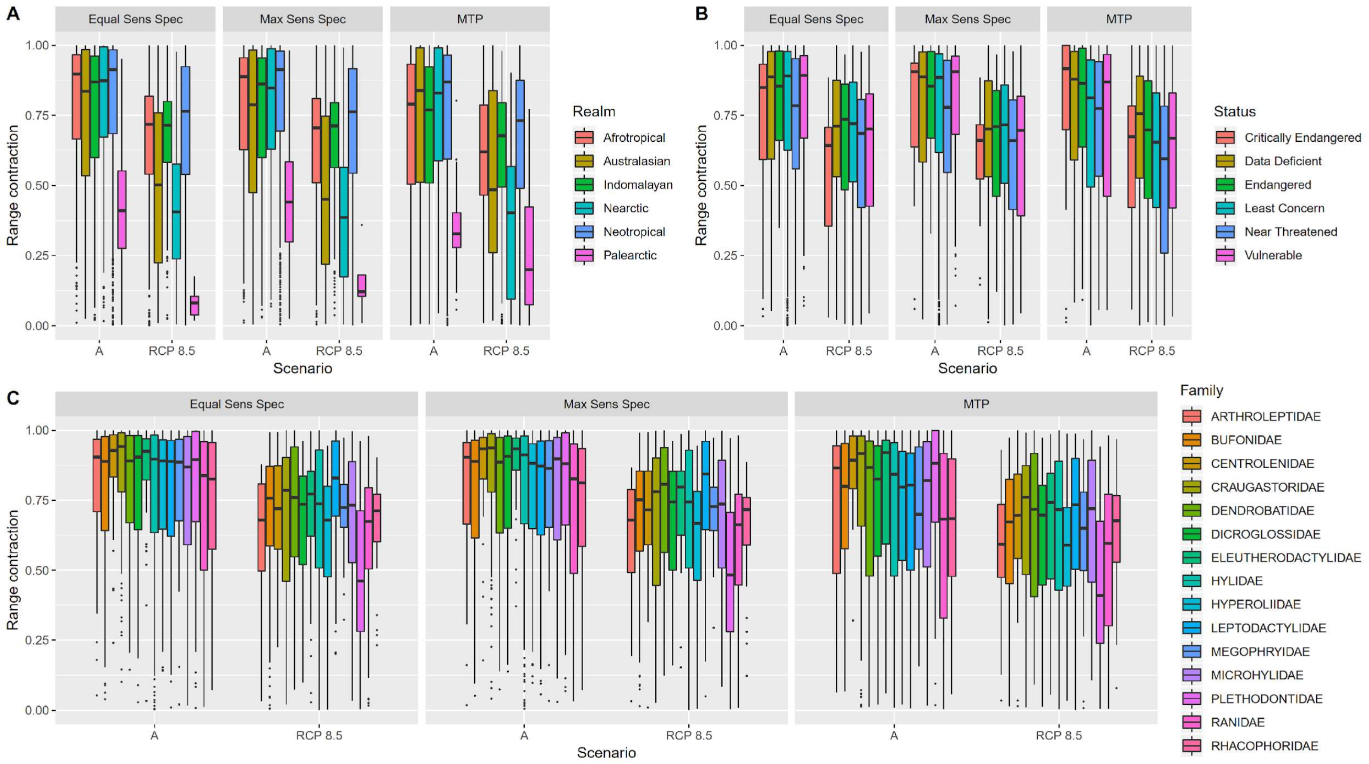

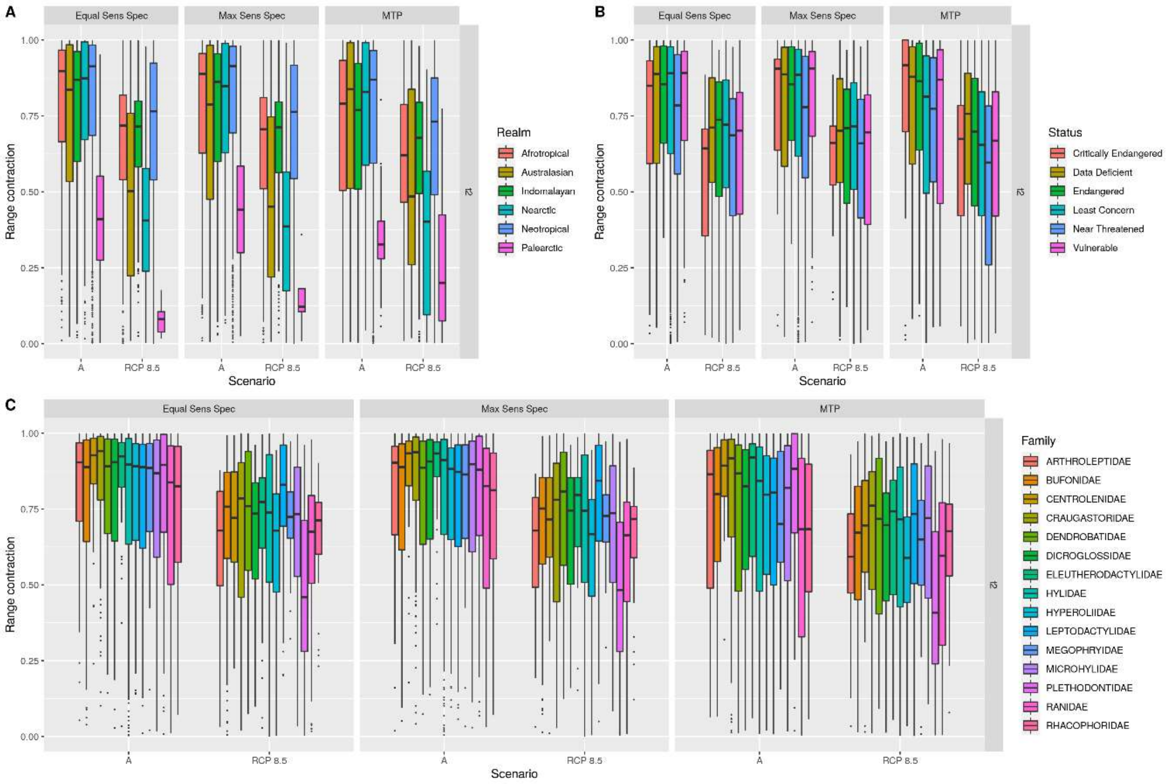

Figure S21. Boxplots of projected range contractions for 2509 amphibian species under a high-warming scenario (RCP 8.5) and the hosing experiment A for 2050 and three threshold criteria to generate presence-absence (i.e., binary) maps. Equal Sens Spec: Equal sensitivity and specificity; Max Sens Spec: Maximizing sensitivity and specificity; and MTP: Minimum

training presence. Results were averaged across three ecological niche modeling algorithms (MaxEnt, BRT, CART; see material and methods) and compared across six biogeographic regions (A), IUCN extinction risk status (B) and for the most diverse amphibian families (i.e., those with more than 100 species; C).

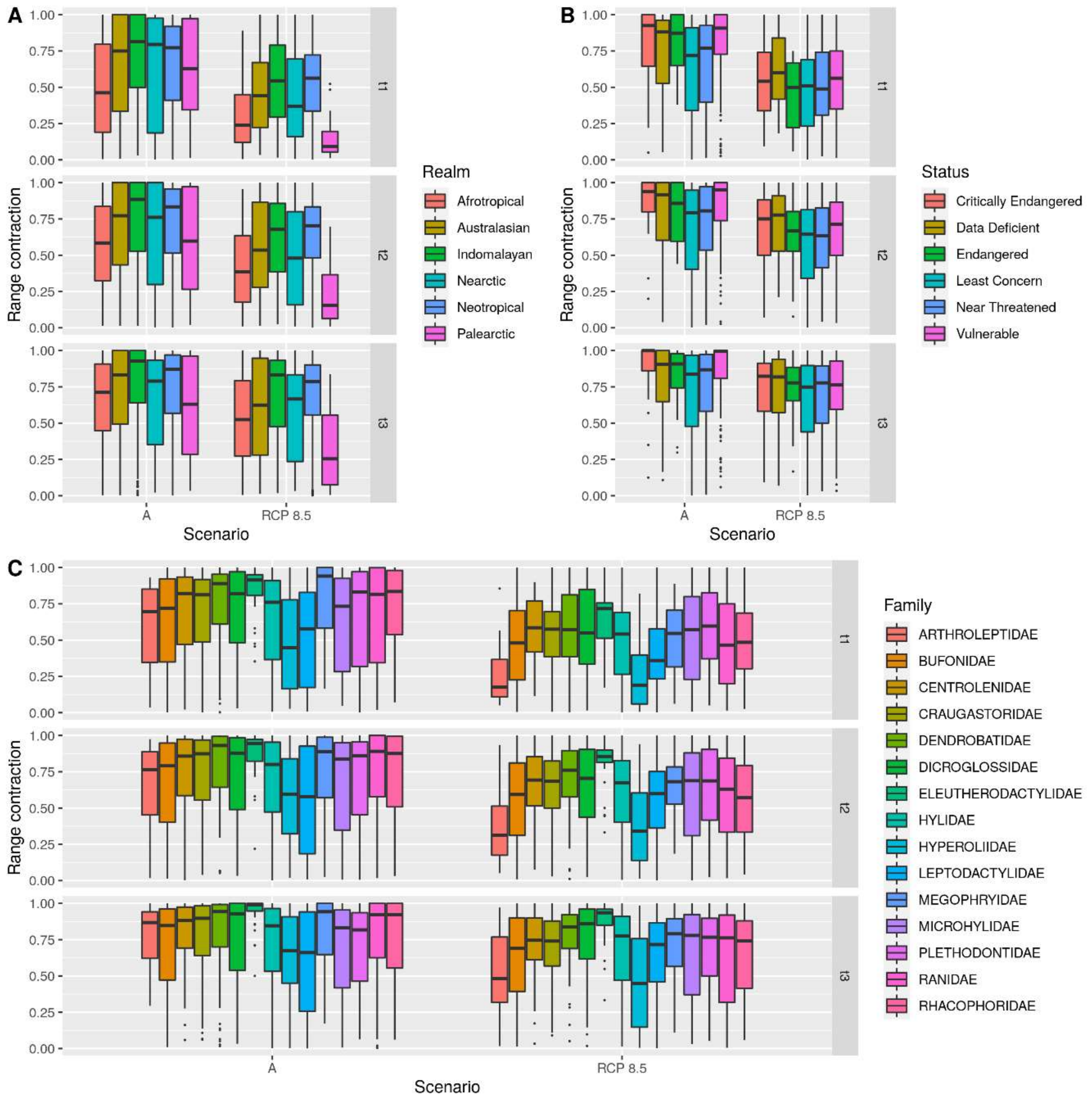

Figure S22. Boxplots of projected range contractions for 2509 amphibian species under a high-warming scenario (RCP 8.5) and the hosing experiment A for three time horizons (t1=2030, t2=2050, t3=2070) using a coarser-grain size (1°). Range contractions are expressed as proportions and are shown only for a full dispersal scenario. Results were averaged across three ecological niche modeling algorithms (MaxEnt, BRT, CART; see material and methods) and compared across six biogeographic regions (A), IUCN extinction risk status (B) and for the most diverse amphibian families (i.e., those with more than 100 species; C).

Our results suggest that impacts of a thermohaline weakening are severe across the three time periods examined (i.e., 2030, 2050, 2070), levels of freshwater discharge (i.e., 0.11 to 0.68 Sv; 1 Sv = 10^6 m^3/s), biogeographical realms, and two dispersal scenarios (Figure 1). Comparisons between projected range losses from the Greenland ice sheet melting simulations and a control simulation reveal substantial increases in projected range losses are expected from these catastrophic scenarios (Figure 1). In addition, we note that endemic species from some biogeographical realms were more susceptible to range contractions. These regions include Palearctic, Nearctic and Australasian, although with a slight variation between model algorithms. The severity of the hosing experiments on a high-warming scenario was evaluated across the conservation status of amphibian species using the extinction risk ranking from IUCN[20]. Some studies have provided evidence that life history and geographical range traits that predict extinction risk are useful as indicators to anticipate vulnerability to climate change[21]. Many of these categories for amphibian species were defined based on geographical range size, population density or relative abundance and potential and direct human stressors. We evaluated the percentage of range contractions across IUCN categories from critically endangered (CR) to least concern (LC) (Figure 1). Threatened species (i.e., species classified as critically endangered, endangered, and vulnerable) will suffer more substantial contractions across hosing experiment scenarios than non-threatened species (i.e., least concern; Figure 1). These results suggest that species with small geographical ranges and less abundant in the wild will be more affected by a rapid ice sheet melting affecting global climate patterns.

S3.5 Comparisons of projected range contractions between scenarios and time horizons

We found significant differences between all four hosing experiments and the control one. The differences were broadly similar either under a full dispersal or no dispersal scenario (Table S4). Full dispersal refers to a scenario where species can occupy suitable climates across a biogeographical realm. In contrast, no dispersal refers to the lack of ability to colonize suitable climates outside its distributional area (Table S4).

Table S4. Comparisons of range contractions between scenarios using an ANOVA test. Control simulation covers the period 2006-2100; A to D denote the four hosing experiments with different levels of freshwater discharge into the North Atlantic Ocean over the period 2020 to 2070. Test results were broadly similar across different niche algorithms and thus only results for the Maxent algorithm are shown.

**Dispersal scenario**	**Comparisons**	**z-value**	**p-value**
**Full dispersal**	RCP 8.5 vs. A (0.11 Sv)	**30.396**	**< 0.001**
	RCP 8.5 vs. B (0.22 Sv)	**29.941**	**< 0.001**
	RCP 8.5 vs. C (0.34 Sv)	**29.353**	**< 0.001**
	RCP 8.5 vs. D (0.68 Sv)	**31.496**	**< 0.001**
	A vs. B	-0.476	0.990
	A vs. C	-0.994	0.858
	A vs. D	1.118	0.797
	B vs. C	-0.519	0.985
	B vs. D	1.594	0.501
	C vs. D	2.109	0.216
**No dispersal**	RCP 8.5 vs. A (0.11 Sv)	**15.656**	**< 0.001**
	RCP 8.5 vs. B (0.22 Sv)	**16.740**	**< 0.001**
	RCP 8.5 vs. C (0.34 Sv)	**15.295**	**< 0.001**
	RCP 8.5 vs. D (0.68 Sv)	**18.869**	**< 0.001**
	A vs. B	1.080	0.817
	A vs. C	-0.362	0.996
	A vs. D	**3.207**	**0.012**
	B vs. C	-1.442	0.600
	B vs. D	2.213	0.209
	C vs. D	**3.569**	**0.003**

S3.6 Geographical patterns of amphibian species losses and gains under full dispersal and no dispersal scenarios.

Species richness maps were generated based on the stacking of presence-absence maps for each species in each biogeographical realm under current climatic conditions and for each climate change scenario. We estimated the percentage of species loss and species gains by each pixel using these species richness maps.

The percentage of species loss were calculated based on the total number of current species richness and how many species were lost in each pixel under each of the climate change scenarios (Figure S23-S29). The geographical patterns of species richness were calculated separately for each ecological niche modeling algorithm (Figures S23-S27) and then were averaged across algorithms to explicitly incorporate this uncertainty source (Figures 2, S28-S29). Similarly, the percentage of species gains (Figures S34-S35) were calculated based on the total number of current species richness and how many species were gained in each pixel under each of the climate change scenarios.

Under a full dispersal scenario, the percentage of projected species losses varies extensively across geography but there are strong similarities across the hosing experiments and temporal horizons, except for 2070 and the most extreme hosing experiment (0.68 sv; Figures S24-S27) in which species loss was much more severe. All realms would be affected by several local extinctions. These results suggest that the potential impacts of climate change plus a thermohaline shutdown would be non-stationary and widespread across regions. We note that the projected local extinctions would be more severe in the Palearctic, northwestern Nearctic, north of India, east of Africa, and southeastern Australia (Figure 2 and Figure S23-S29). The amphibian assemblages inhabiting these regions would be more impacted by severe range contractions driven by a catastrophic climate change event. To further illustrate the effects of additional weakening of AMOC, figures S30 to S32 show the difference in the geographical patterns of percentage of species losses between RCP8.5 and each one of the hosing experiments. These figures reveal the highly non-linear response of the amphibian assemblages to these freshwater discharge scenarios and suggest the possibility of a tipping point behavior.

The estimated percentage of species loss is the result of the interplay between changes in climate and the species response to such changes. As discussed in section S2 and in previous paragraphs, changes in climate are spatially very heterogeneous, particularly under the hosing experiments. Similarly, the responses to changes in climate are idiosyncratic and the same climatic variation can produce substantially different impacts on the 2,509 amphibian species considered in this paper. Figure S30 illustrates the spatial heterogeneity of changes in climate and of impacts on amphibian species, as well as their association at the grid scale for experiment D (0.68 sv) and the control scenario (RCP 8.5). As is shown in this figure, large changes in climate are typically associated with high percentage of species loss. However, due to the diversity of species and their sensitivity, in some cases large (small) losses can occur for relatively small (large) changes in climate. The diversity in changes in climate at the grid cell level and the idiosyncratic responses of the amphibian species modelled makes it difficult to extract general conclusions about what combinations of the bioclimatic variables drive the overall impacts in each realm.

Under a no dispersal scenario, the percentage of species losses are consequently more drastic (Figure S34-S36). The species losses under a high-emission scenario for the first time horizon are less severe than for the hosing scenarios (Fig. S34-a vs. Fig. S34-c). The differences are not very large across time horizons for experiment D (Figure S34-b vs. Fig. S34-d). In addition, the species losses were highly similar across hosing scenarios and time periods (Fig. S36). However, we consider that these no dispersal scenarios likely are unrealistic because they reduce to zero the ability of species to track their climate requirements through geography and the projected species losses might be extremely inflated. Figure S37 shows the percentage of range expansions in each one of the scenarios. Range expansions tend to be reduced and homogeneous across all climate change scenarios.

Finally, the geographical patterns of percentage of species gains (figures S38-S39) show that species gains in regional assemblages are more frequent and pervasive in a high-emissions scenario (RCP8.5) than in hosing experiments (Figure S34). Although the Neotropical region tends to exhibit some gains of species in comparison to other regions, the highest gains were toward temperate regions (e.g., the United States and Canada). Although these maps show the opposite patterns of species losses, these gains implicate that any species can colonize a given cell from any part of each realm and this can be unrealistic for many species due to limited dispersal. A further study will be necessary to evaluate properly the turnover in species richness across time horizons and scenarios using recently developed metrics of temporal turnover[22].

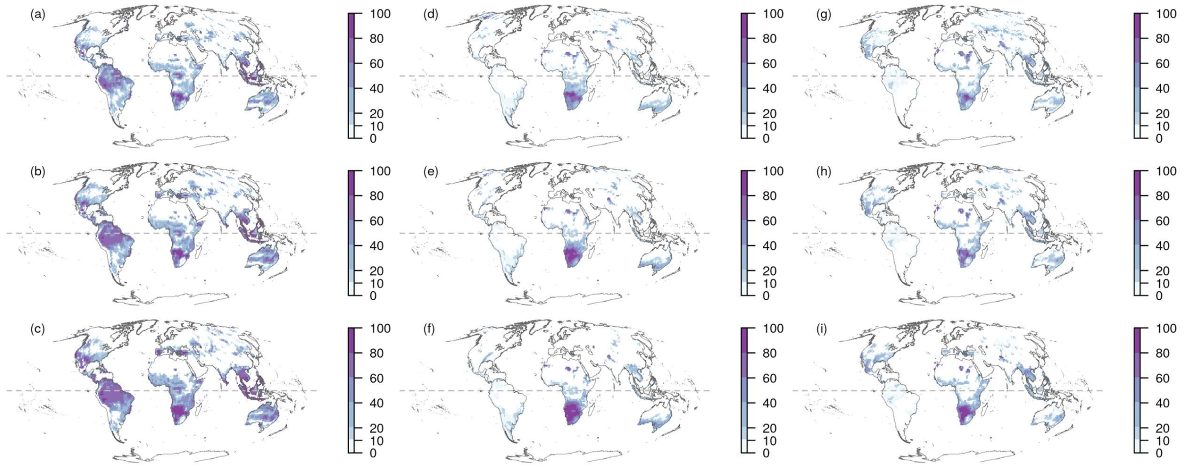

Figure S23. Global geographical patterns of percentage of species loss for a high-emissions scenario (RCP 8.5) for three niche model algorithms and three temporal horizons under a full dispersal scenario. a) MaxEnt 2030; b) MaxEnt 2050; c) MaxEnt 2070; d) BRT 2030; e) BRT 2050; f) BRT 2070; g) CART 2030; h) CART 2050; i) CART 2070.

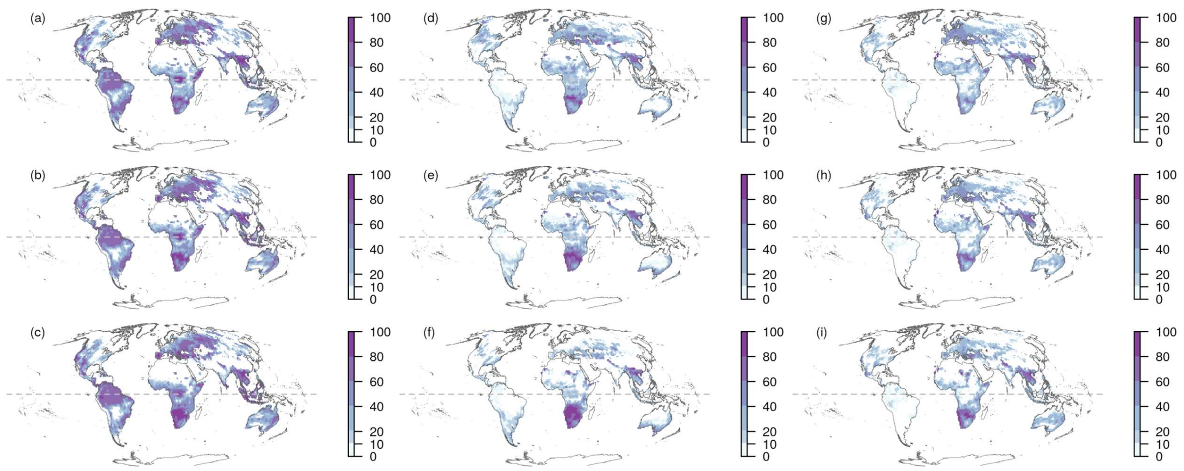

Figure S24. Global geographical patterns of percentage of species loss for the hosing experiment A and for three niche model algorithms and three temporal horizons under a no dispersal scenario. a) MaxEnt 2030; b) MaxEnt 2050; c) MaxEnt 2070; d) BRT 2030; e) BRT 2050; f) BRT 2070; g) CART 2030; h) CART 2050; i) CART 2070.

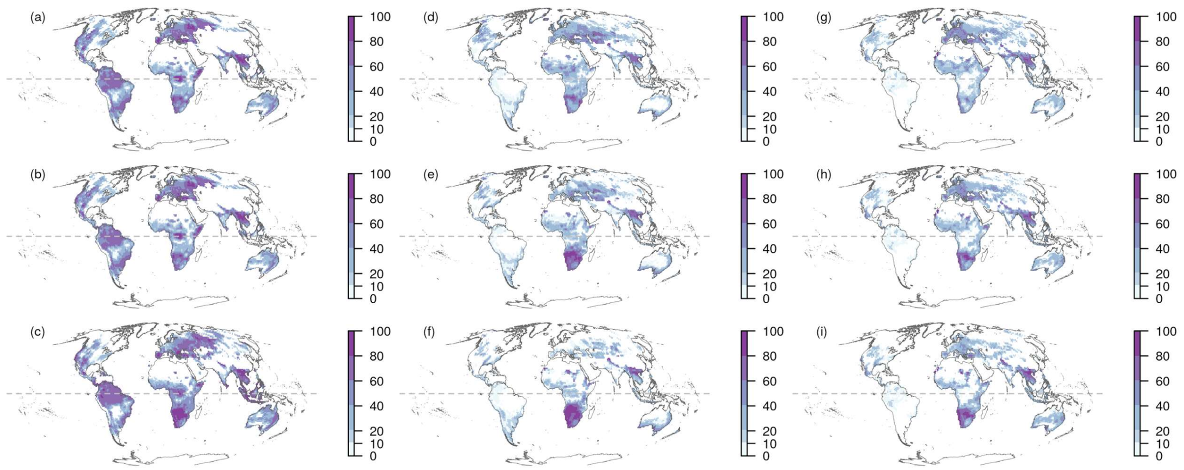

Figure S25. Global geographical patterns of percentage of species loss for the hosing experiment B and for three niche model algorithms and three temporal horizons under a full dispersal scenario. a) MaxEnt 2030; b) MaxEnt 2050; c) MaxEnt 2070; d) BRT 2030; e) BRT 2050; f) BRT 2070; g) CART 2030; h) CART 2050; i) CART 2070.

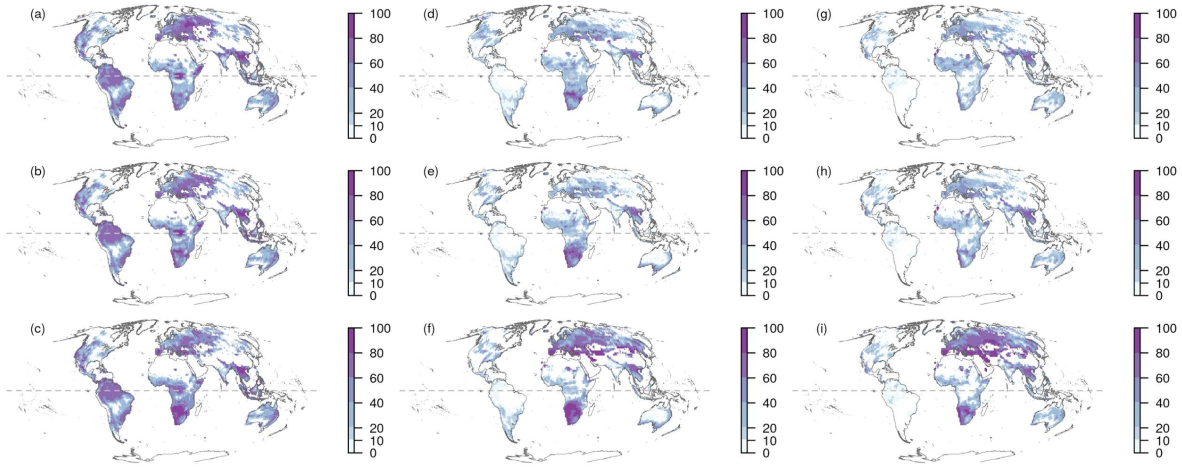

Figure S26. Global geographical patterns of percentage of species loss for the hosing experiment C and for three niche model algorithms and three temporal horizons under a full dispersal scenario. a) MaxEnt 2030; b) MaxEnt 2050; c) MaxEnt 2070; d) BRT 2030; e) BRT 2050; f) BRT 2070; g) CART 2030; h) CART 2050; i) CART 2070.

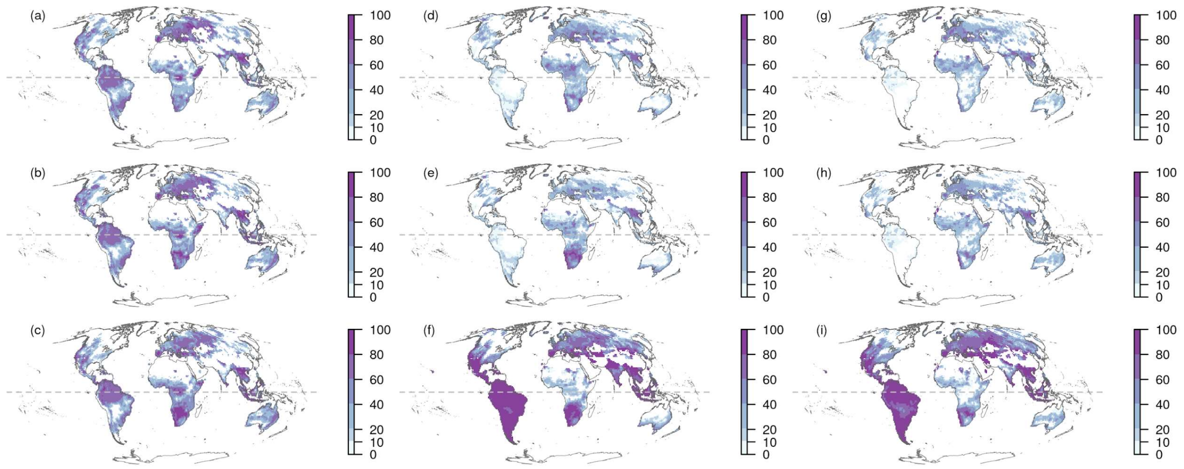

Figure S27. Global geographical patterns of percentage of species loss for the hosing experiment D and for three niche model algorithms and three temporal horizons under a full dispersal scenario. a) MaxEnt 2030; b) MaxEnt 2050; c) MaxEnt 2070; d) BRT 2030; e) BRT 2050; f) BRT 2070; g) CART 2030; h) CART 2050; i) CART 2070.

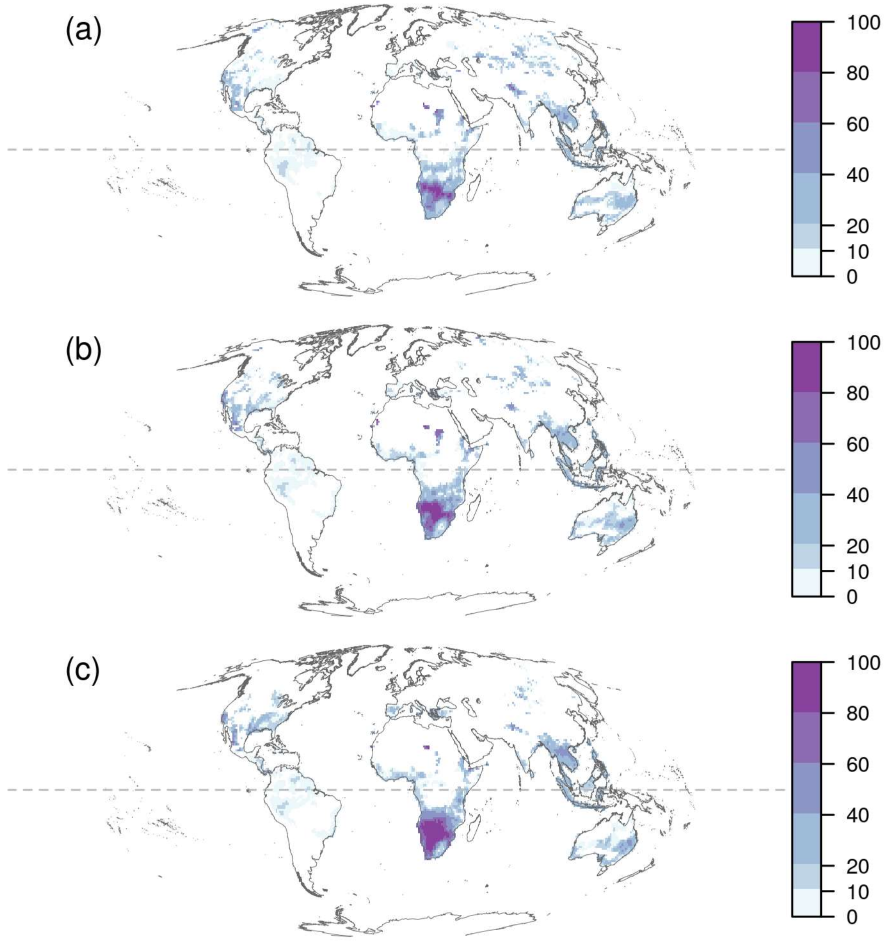

Figure S28. Global geographical patterns of percentage of species loss for a high-warming scenario (RCP 8.5), averaged across three niche model algorithms for a full dispersal scenario. a) 2030; b) 2050; c) 2070.

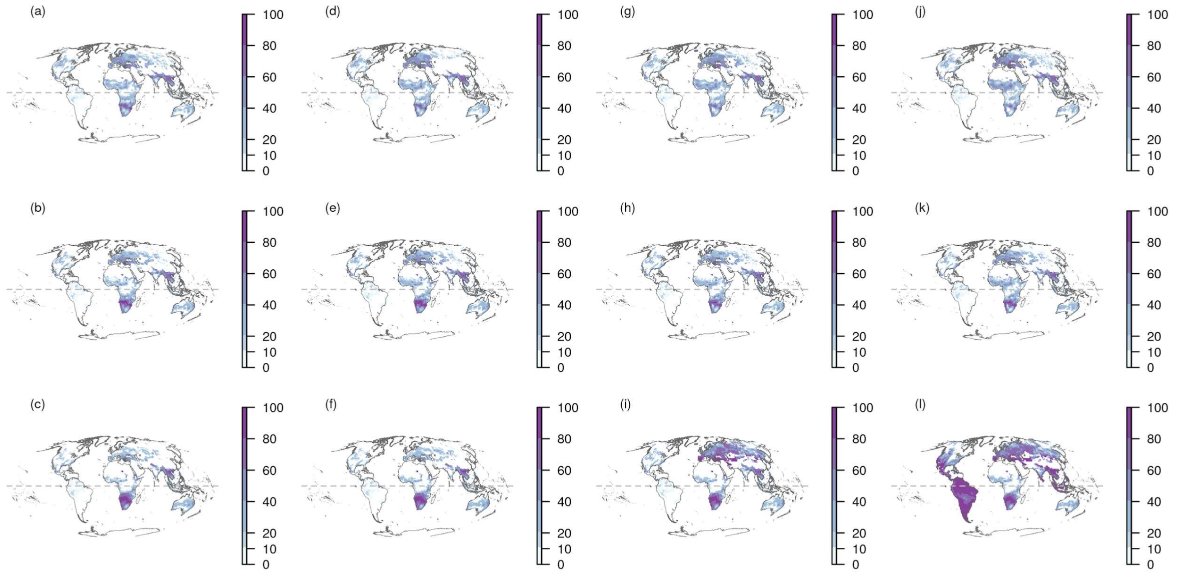

Figure S29. Global geographical patterns of percentage of species loss for all hosing experiments averaged across three niche model algorithms under a full dispersal. a) simulation A for 2030; b) simulation A for 2050; c) simulation A for 2070; d) simulation B for 2030; e) simulation B for 2050; f) simulation B for 2070; g) simulation C for 2030; h) simulation C for 2050; i) simulation C for 2070; j) simulation D for 2030; k) simulation D for 2050; l) simulation D for 2070.

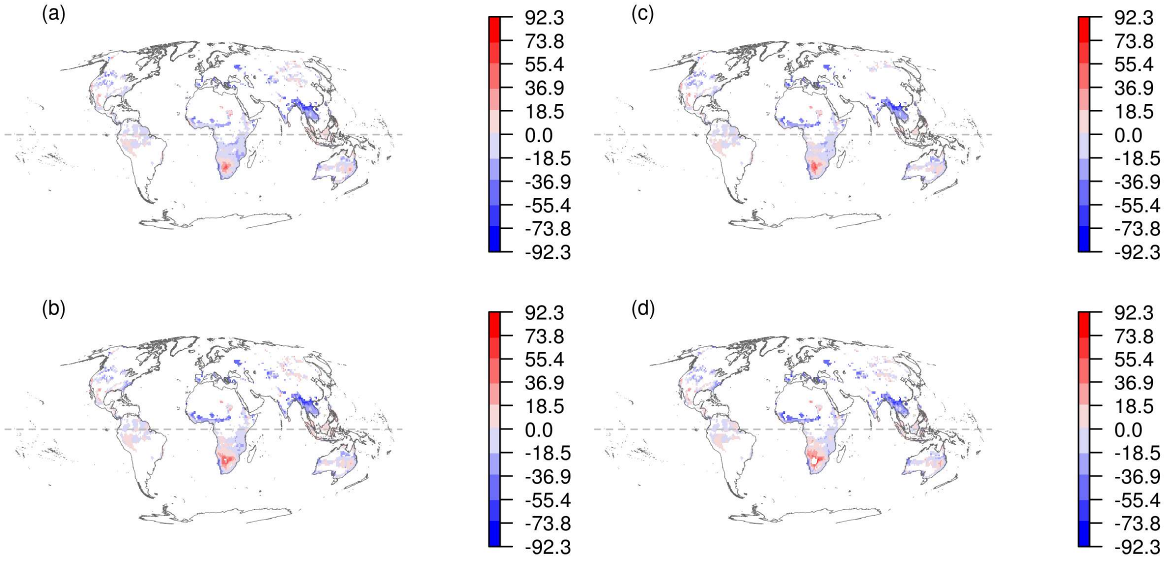

Figure S30. Differences in percentage values of species loss between the control reference high-emission scenario (RCP8.5) and the four hosing experiments for 2030. a) RCP8.5 vs. experiment A; b) RCP8.5 vs. experiment B; c) RCP8.5 vs. experiment C; d) RCP8.5 vs. experiment D. Red values indicates that percentage differences in species losses were higher in a high-emission scenario (RCP8.5) than a given hosing experiment. By contrast, blue values indicate that species losses were higher in a given hosing experiment than in the control high-emission scenario (RCP8.5).

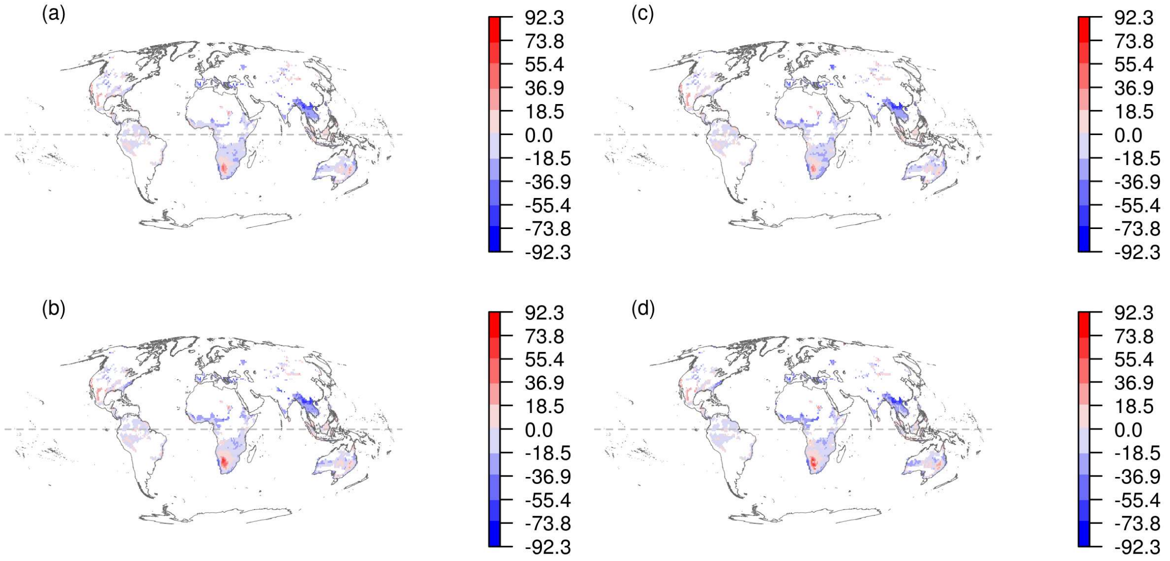

Figure S31. Differences in percentage values of species loss between the control reference high-emission scenario (RCP8.5) and the four hosing experiments for 2050. a) RCP8.5 vs. experiment A; b) RCP8.5 vs. experiment B; c) RCP8.5 vs. experiment C; d) RCP8.5 vs. experiment D. Red values indicates that species losses were higher in a high-emission scenario (RCP8.5) than a given hosing experiment. By contrast, blue values indicate that species losses were higher in a given hosing experiment than in the control high-emission scenario (RCP8.5).

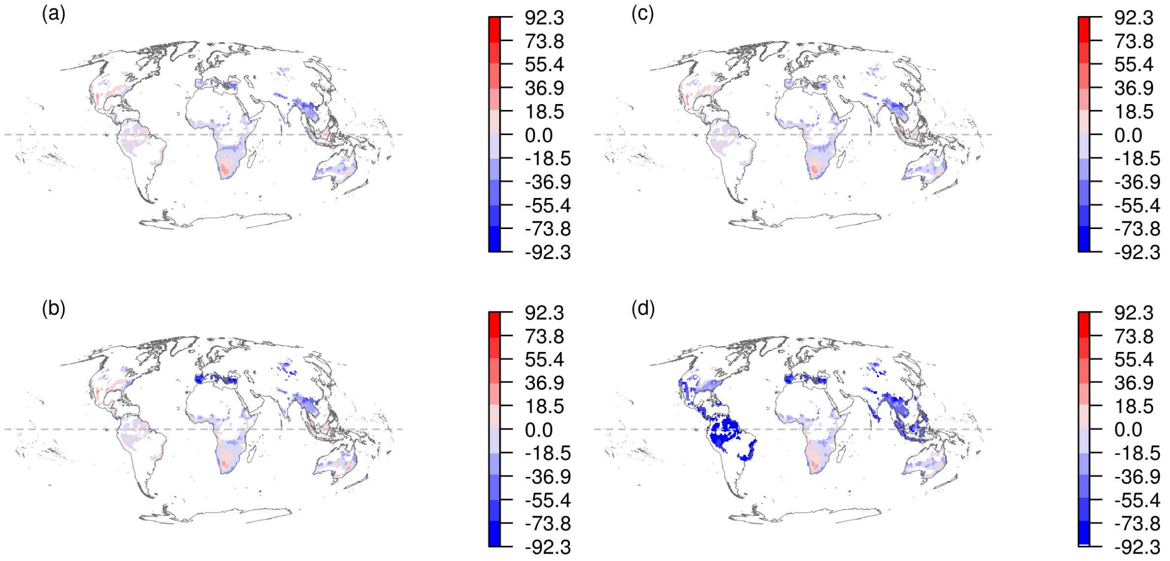

Figure S32. Differences in percentage values of species loss between the control reference high-emission scenario (RCP8.5) and the four hosing experiments for 2070. a) RCP8.5 vs. experiment A; b) RCP8.5 vs. experiment B; c) RCP8.5 vs. experiment C; d) RCP8.5 vs. experiment D. Red values indicates that species losses were higher in a high-emission scenario (RCP8.5) than a given hosing experiment. By contrast, blue values indicate that species losses were higher in a given hosing experiment than the control high-emission scenario (RCP8.5).

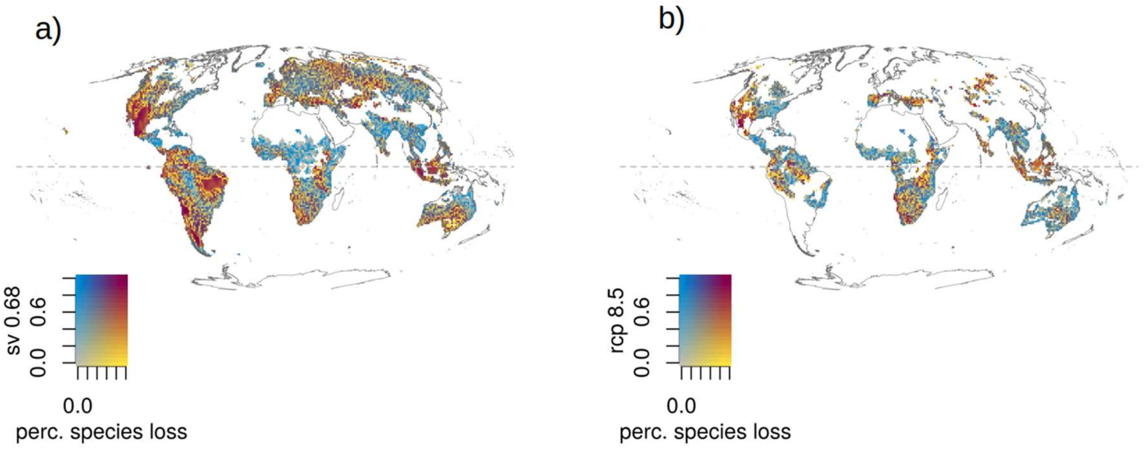

Figure S33. Association between percentage of species loss and climatic departures in 2070. Panels a) and b) show the association between percentage of species loss and climatic departures for the hosing experiment D and RCP8.5 control reference simulation, respectively. Color gradient from: 1) grey to blue denotes increasing levels of climatic departure; 2) grey to yellow indicates increasing percentage of species loss; 3) grey to red denotes both increasing levels of climate departure and percentage of species loss.

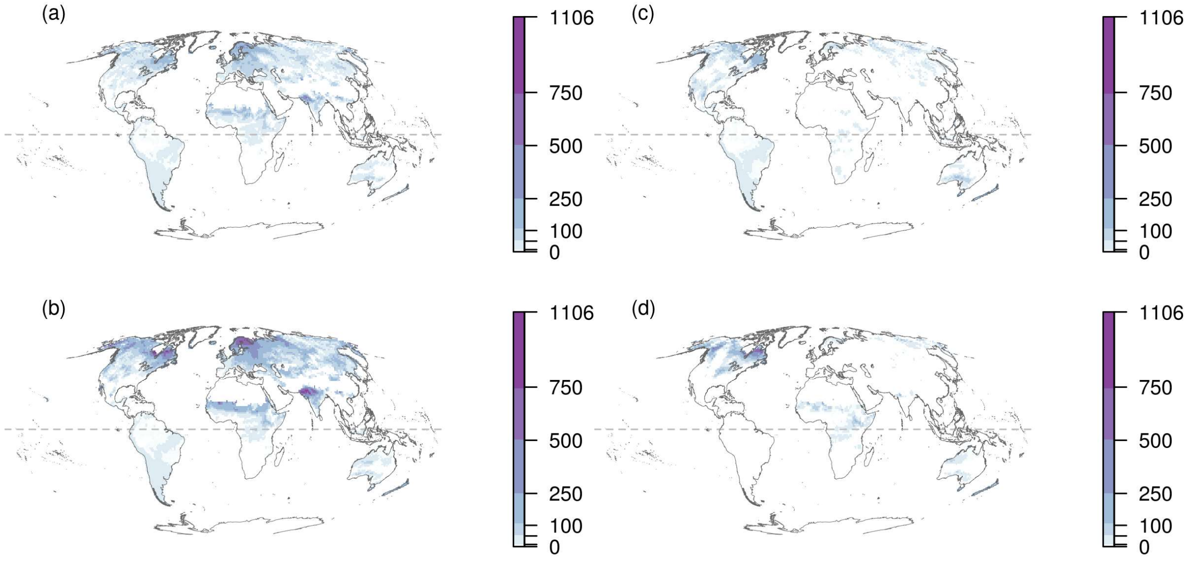

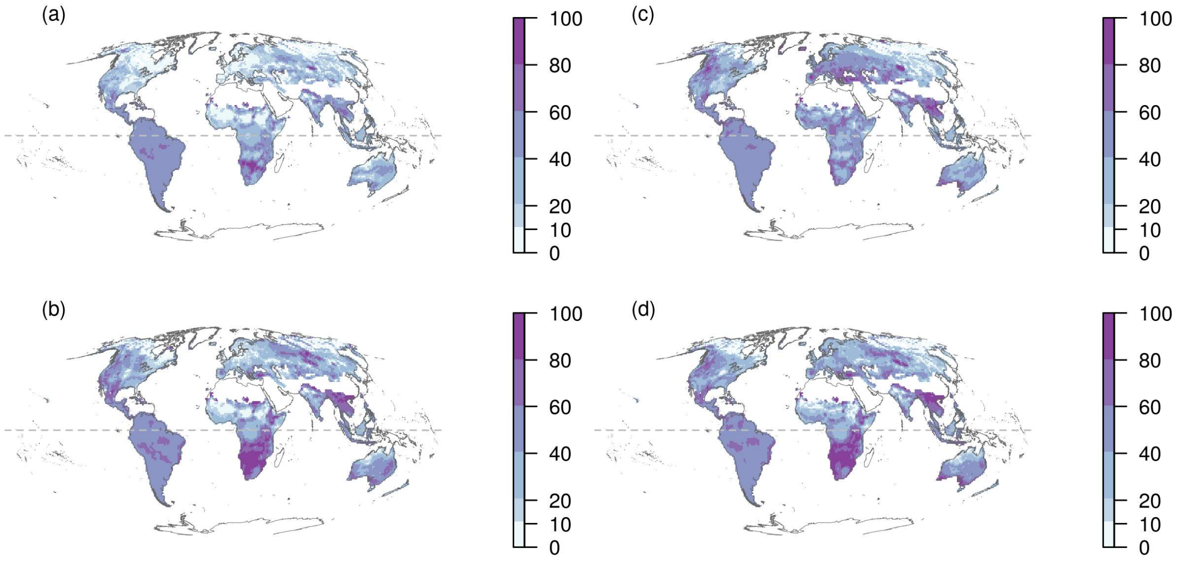

Figure S34. Geographical patterns of percentage of species loss under a no dispersal scenario across six biogeographical regions (Afrotropical, Australasian, Indomalaya, Nearctic, Neotropical and Palearctic) for different climate change scenarios. (a) and (b) correspond to mapping of the percentage of species loss per pixel (1°) under the control reference high-warming scenario (RCP 8.5) for 2030 and 2070, respectively. (c) and (d) correspond to mapping of the percentage of species loss under the hosing experiment D (0.68-Sv; 1 Sv = 10^6 m^3/s) for 2030 and 2070, respectively. The geographical patterns were calculated averaging the results from three niche modeling algorithms (Maxent, BRT, CART) to account for model uncertainty explicitly in the mapping of species loss across the globe.

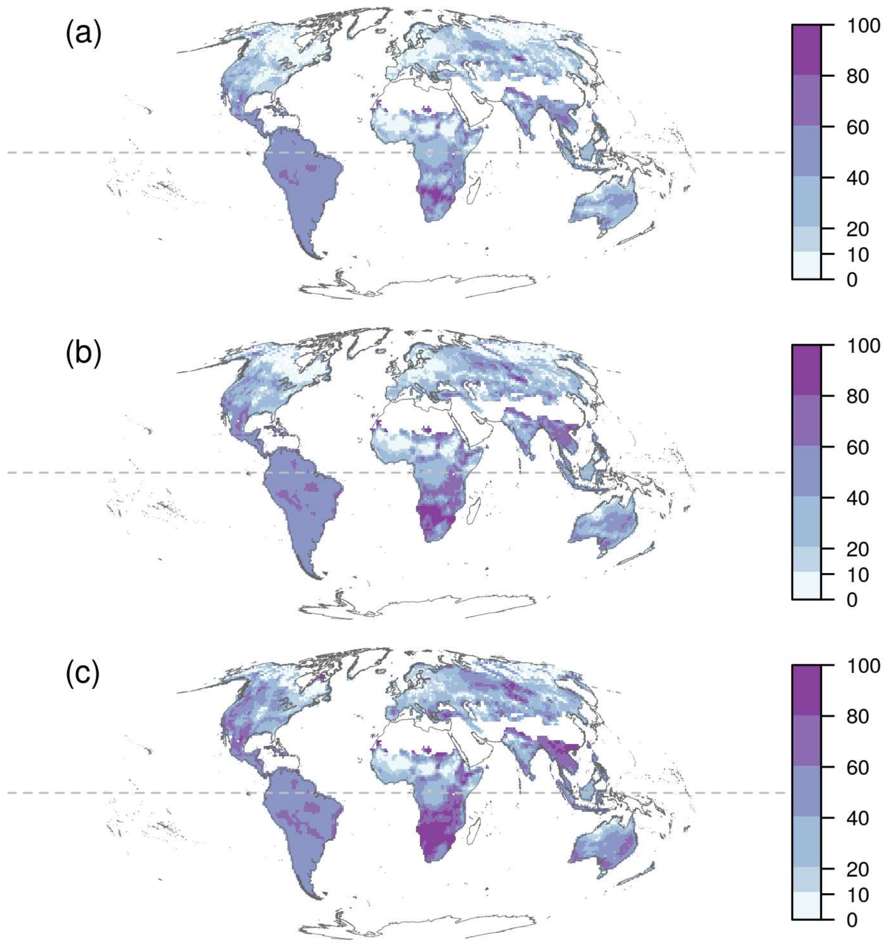

Figure S35. Global geographical patterns of percentage of species loss under a no dispersal scenario for the control reference high-warming scenario (RCP 8.5), averaged across three niche model algorithms. a) 2030; b) 2050; c) 2070.

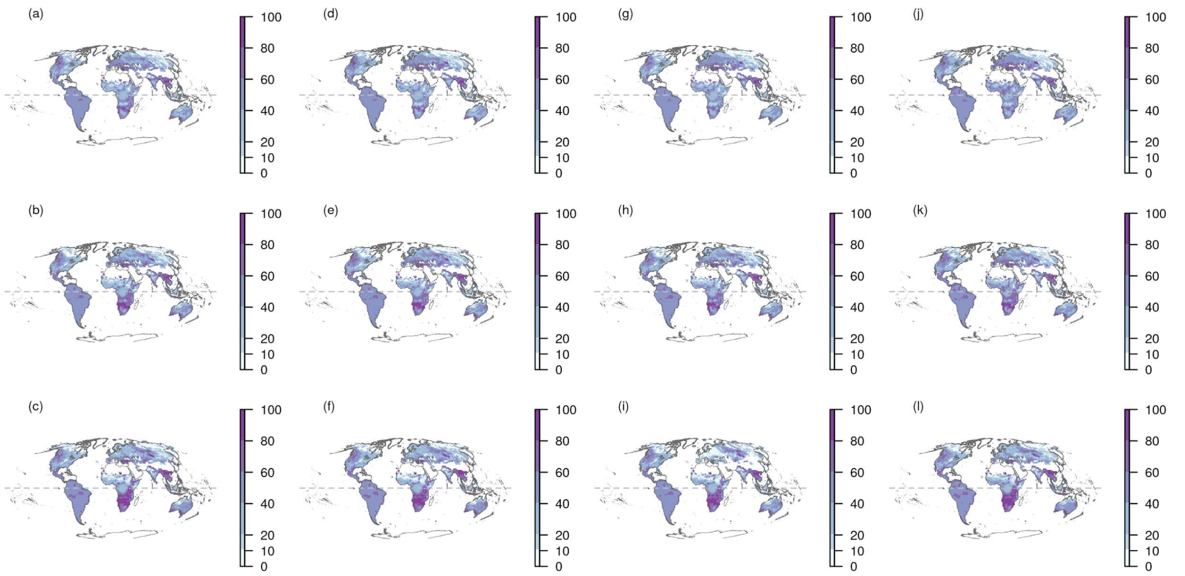

Figure S36. Global geographical patterns of percentage of species loss under a no dispersal scenario for all hosing experiments averaged across three niche model algorithms. a) simulation A for 2030; b) simulation A for 2050; c) simulation A for 2070; d) simulation B for 2030; e) simulation B for 2050; f) simulation B for 2070; g) simulation C for 2030; h) simulation C for 2050; i) simulation C for 2070; j) simulation D for 2030; k) simulation D for 2050; l) simulation D for 2070.

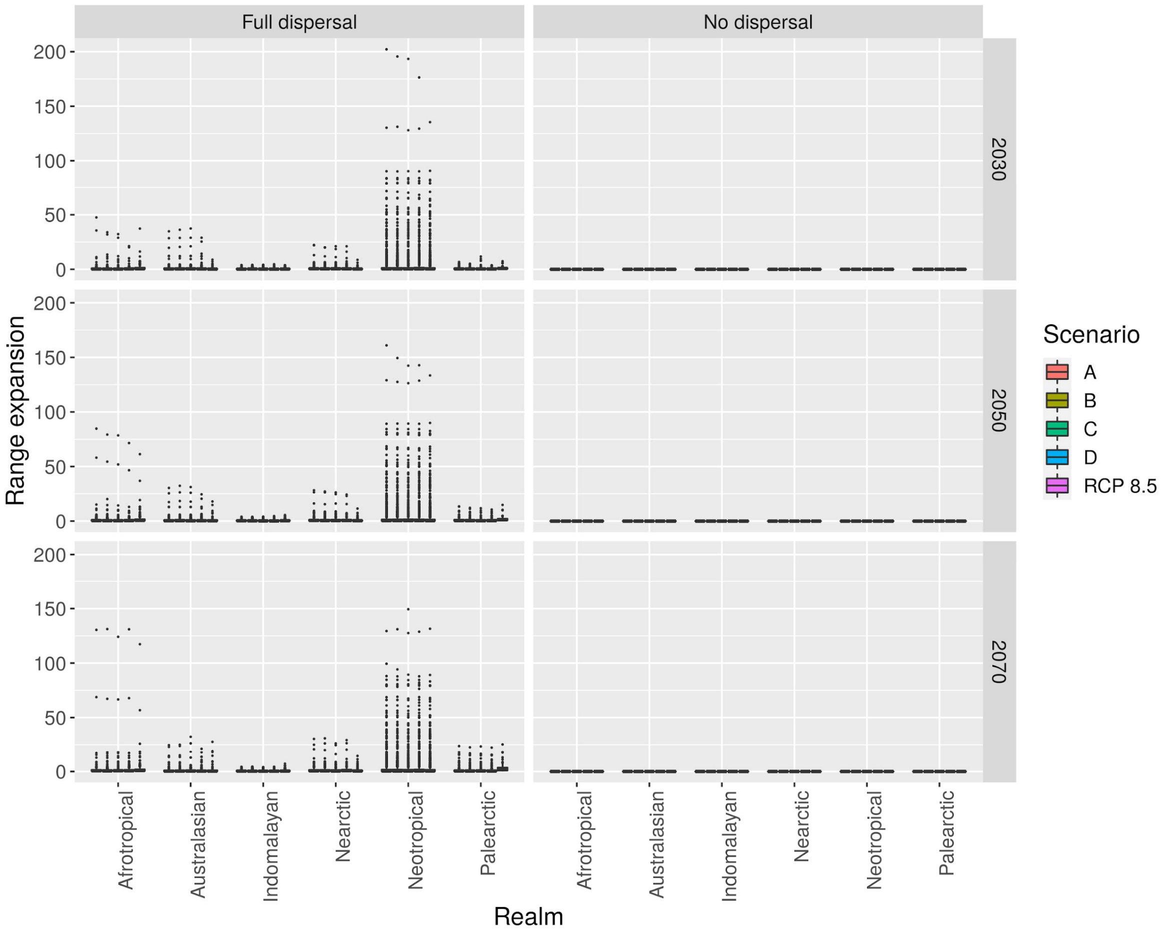

Figure S37. Boxplots of projected range expansions for amphibian species expanding their ranges under the control reference high-emission scenario (RCP 8.5; reference scenario) and the same scenario including four different hosing rates of0.11, 0.22, 0.34, and 0.68-Sv of freshwater (1 Sv = 10^6 m^3/s) labeled as A, B, C, and D, respectively. The number of species expanding their ranges varied across scenarios, time horizon, niche modeling algorithms and realms (Table S2-S3). Range expansions are expressed in proportional terms and are shown for two dispersal scenarios (full dispersal and no dispersal). Results were averaged across three ecological niche modeling algorithms (MaxEnt, BRT, CART; see material and methods) and compared across six biogeographic regions.

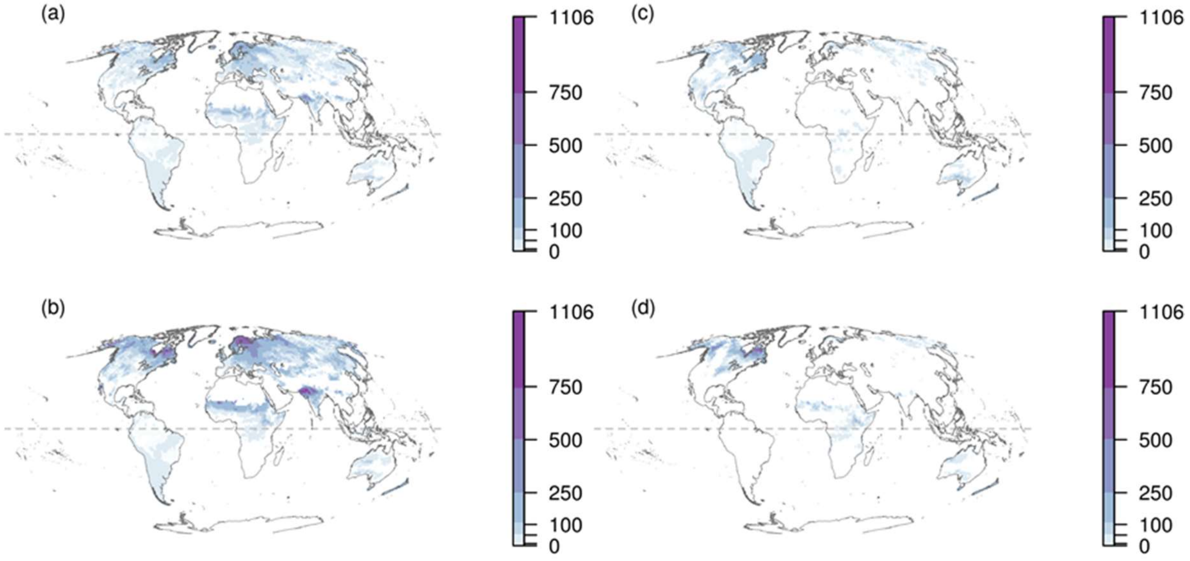

Figure S38. Geographical patterns of percentage of species gains under a full dispersal scenario across six biogeographical regions (Afrotropical, Australasian, Indomalaya, Nearctic, Neotropical and Palearctic) for different climate change scenarios. (a) and (b) correspond to mapping of the percentage of species gains per pixel (1°) under the control reference high-warming scenario (RCP 8.5) for 2030 and 2070, respectively. (c) and (d) correspond to mapping of the percentage of species gains under the hosing experiment D (0.68-Sv; 1 Sv = 10^6 m^3/s) for 2030 and 2070, respectively. The geographical patterns were calculated averaging the results from three niche modeling algorithm (Maxent, BRT, CART) to account for model uncertainty explicitly in the mapping of species gains across the globe.

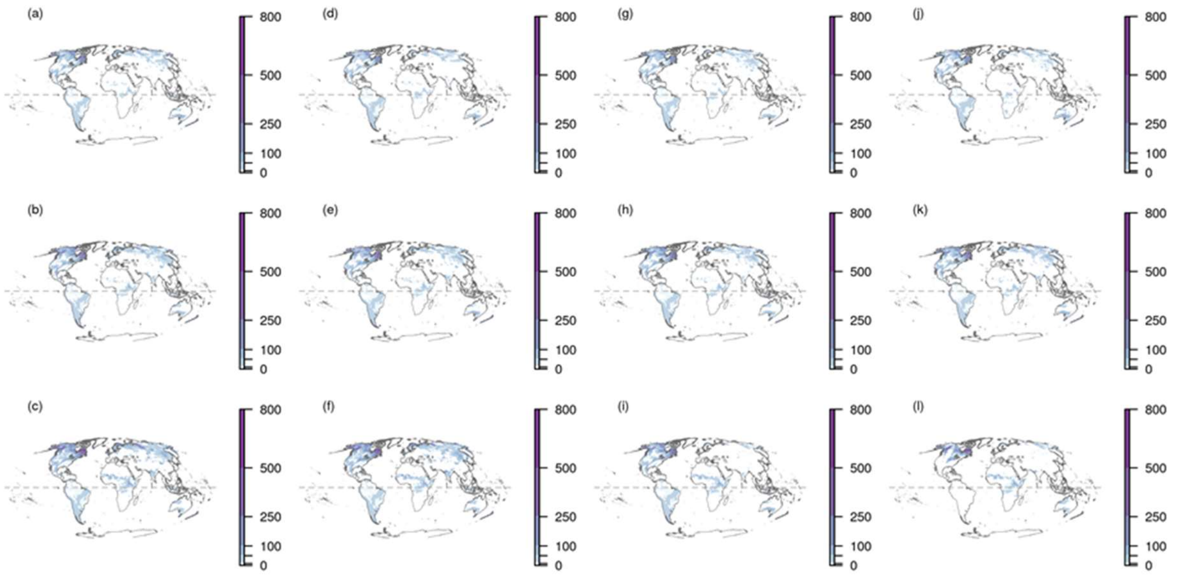

Figure S39. Global geographical patterns of percentage of species gains for all hosing experiments averaged across three niche model algorithms under a full dispersal scenario. a) simulation A for 2030; b) simulation A for 2050; c) simulation A for 2070; d) simulation B for 2030; e) simulation B for 2050; f) simulation B for 2070; g) simulation C for 2030; h) simulation C for 2050; i) simulation C for 2070; j) simulation D for 2030; k) simulation D for 2050; l) simulation D for 2070.

References

1. Dufresne, J. L. *et al.* Climate change projections using the IPSL-CM5 Earth System Model: From CMIP3 to CMIP5. *Clim. Dyn.* **40**, 2123–2165 (2013).

2. Swingedouw, D. *et al.* Decadal fingerprints of freshwater discharge around Greenland in a multi-model ensemble. *Clim. Dyn.* **41**, 695–720 (2013).

3. Defrance, D. *et al.* Consequences of rapid ice sheet melting on the Sahelian population vulnerability. *Proc. Natl. Acad. Sci. U. S. A.* **114**, 6533–6538 (2017).

4. Vellinga, M. & Wood, R. A. Global climatic impacts of a collapse of the atlantic thermohaline circulation. *Clim. Change* **54**, 251–267 (2002).

5. Lenton, T. M. *et al.* Tipping elements in the Earth's climate system. *Proc. Natl. Acad. Sci. U. S. A.* **105**, 1786–93 (2008).

6. Collins, M. & Sutherland, M. Extremes, Abrupt Changes and Managing Risks. in *IPCC Special Report on the Ocean and Cryosphere in a Changing Climate* (eds. Pörtner, H.-O. et al.) 3–63 (In press, 2019).

7. van Vuuren, D. P. *et al.* The use of scenarios as the basis for combined assessment of climate change mitigation and adaptation. *Glob. Environ. Chang.* **21**, 575–591 (2011).

8. Schwalm, C. R., Glendon, S. & Duffy, P. B. RCP8.5 tracks cumulative CO2 emissions. *Proc. Natl. Acad. Sci.* **117**, 202007117 (2020).

9. Munguía, M., Rahbek, C., Rangel, T. F., Diniz-Filho, J. A. F. & Araújo, M. B. Equilibrium of global amphibian species distributions with climate. *PLoS One* **7**, (2012).

10. Ochoa-Ochoa, L. M., Mejía-Domínguez, N. R., Velasco, J. A., Marske, K. A. & Rahbek, C. Amphibian functional diversity is related to high annual precipitation and low precipitation seasonality in the New World. *Glob. Ecol. Biogeogr.* **28**, 1219–1229 (2019).

11. Araújo, M. B. *et al.* Quaternary climate changes explain diversity among reptiles and amphibians. *Ecography (Cop.).* **31**, 8–15 (2008).

12. Thuiller, W., Guéguen, M., Renaud, J., Karger, D. N. & Zimmermann, N. E. Uncertainty in ensembles of global biodiversity scenarios. *Nat. Commun.* **10**, (2019).

13. Oliveira, B. F., Sheffers, B. R. & Costa, G. C. Decoupled erosion of amphibians' phylogenetic and functional diversity due to extinction. *Glob. Ecol. Biogeogr.* **29**, 309–319 (2020).

14. Williams, J. W. & Jackson, S. T. Novel climates, no-analog communities, and ecological surprises. *Front. Ecol. Environ.* **5**, 475–482 (2007).

15. Garcia, R. A., Cabeza, M., Rahbek, C. & Araújo, M. B. Multiple dimensions of

climate change and their implications for biodiversity. *Science* vol. 344 (2014).

16. Fielding, A. H. & Bell, J. F. A review of methods for the assessment of prediction errors in conservation presence/absence models. *Environ. Conserv.* **24**, 38–49 (1997).

17. Allouche, O., Tsoar, A. & Kadmon, R. Assessing the accuracy of species distribution models: Prevalence, kappa and the true skill statistic (TSS). *J. Appl. Ecol.* (2006) doi:10.1111/j.1365-2664.2006.01214.x.

18. Peterson, A. T. *et al. Ecological Niches and Geographic Distributions (MPB-49)*. *Ecological Niches and Geographic Distributions (MPB-49)* (Princeton University Press, 2011). doi:10.23943/princeton/9780691136868.001.0001.

19. Guisan, A., Thuiller, W. & Zimmermann, N. E. *Habitat suitability and distribution models: With applications in R*. *Habitat Suitability and Distribution Models: With Applications in R* (2017). doi:10.1017/ 9781139028271.

20. IUCN. IUCN Red List of Threatened Species. Version 2019-3. *IUCN 2019* http://www.iucnredlist.org (2019).

21. Pearson, R. G. *et al.* Life history and spatial traits predict extinction risk due to climate change. *Nat. Clim. Chang.* **4**, 217–221 (2014).

22. Magurran, A. E. *et al*. Temporal β diversity—A macroecological perspective. *Glob. Ecol. and Biog.* **28**, 1949-1960 (2019).
